# Molecular Evolution of Lysine Biosynthesis in Agaricomycetes

**DOI:** 10.3390/jof8010037

**Published:** 2021-12-31

**Authors:** Zili Song, Maoqiang He, Ruilin Zhao, Landa Qi, Guocan Chen, Wen-Bing Yin, Wei Li

**Affiliations:** 1 State Key Laboratory of Mycology, Institute of Microbiology, Chinese Academy of Sciences, Beijing 100101, China; sqsfswgcszl@163.com (Z.S.); hemaoqiangleo@gmail.com (M.H.); zhaorl@im.ac.cn (R.Z.); 2 Savaid Medical School, University of Chinese Academy of Sciences, Beijing 100049, China; 3 Henan Academy of Science Institute of Biology, Zhengzhou 450008, China; 2012202024@njau.edu.cn (L.Q.); qbsxwh@163.com (G.C.)

**Keywords:** molecular evolution, lysine, biosynthetic enzymes, mushroom, Agaricomycetes

## Abstract

As an indispensable essential amino acid in the human body, lysine is extremely rich in edible mushrooms. The α-aminoadipic acid (AAA) pathway is regarded as the biosynthetic pathway of lysine in higher fungal species in Agaricomycetes. However, there is no deep understanding about the molecular evolutionary relationship between lysine biosynthesis and species in Agaricomycetes. Herein, we analyzed the molecular evolution of lysine biosynthesis in Agaricomycetes. The phylogenetic relationships of 93 species in 34 families and nine orders in Agaricomycetes were constructed with six sequences of LSU, SSU, ITS (5.8 S), RPB1, RPB2, and EF1-α datasets, and then the phylogeny of enzymes involved in the AAA pathway were analyzed, especially homocitrate synthase (HCS), α-aminoadipate reductase (AAR), and saccharopine dehydrogenase (SDH). We found that the evolution of the AAA pathway of lysine biosynthesis is consistent with the evolution of species at the order level in Agaricomycetes. The conservation of primary, secondary, predicted tertiary structures, and substrate-binding sites of the enzymes of HCS, AAR, and SDH further exhibited the evolutionary conservation of lysine biosynthesis in Agaricomycetes. Our results provide a better understanding of the evolutionary conservation of the AAA pathway of lysine biosynthesis in Agaricomycetes.

## 1. Introduction

**Figure 1:**
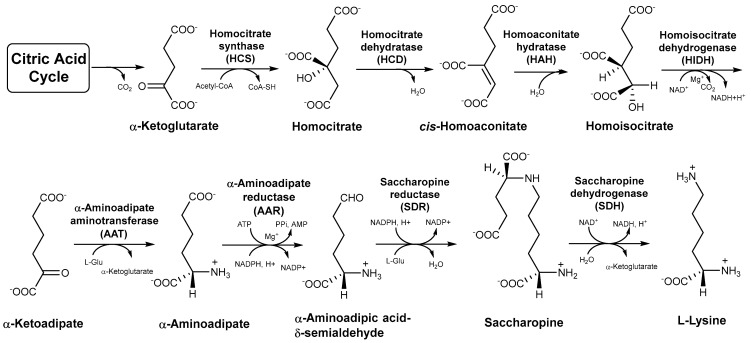
The α−aminoadipate (AAA) pathway of lysine biosynthesis in higher fungi.

Lysine is an essential amino acid required for normal growth and development in the human body [1,2]. Lack of lysine will not only impair protein synthesis but also seriously affect the organismal immune and central nervous systems [3,4]. Lysine exhibits promising application prospects in the food, chemical synthesis, cosmetic, and pharmaceutical industries. Edible wild fungi in Agaricomycetes are important sources of amino acids and serve as excellent food sources of lysine for humans [5,6]. There are three perspectives recognized in the origin and evolution of lysine metabolic pathways, including the retro-evolution hypothesis, forward development hypothesis, and enzymatic recruitment hypothesis [7,8,9]. The enzymatic recruitment hypothesis of lysine biosynthesis is widely accepted based on the available research. Lysine is the only amino acid among protein-derived amino acids that is involved in two different synthetic pathways in organisms [2,10]. In bacteria, lower fungi, and green plants, the diaminopimelate (DAP) pathway of lysine biosynthesis begins with aspartate semialdehyde and pyruvate. The α-aminoadipate (AAA) pathway of lysine biosynthesis is found in higher fungi and euglenoids with α-ketoglutarate from citric acid cycle as the precursor [2,9]. Moreover, the intermediates in the AAA pathway are incorporated into secondary metabolites with biological activities such as penicillin, slaframine, and swainsonine [10]. There are seven intermediates and eight enzymes involved in the AAA pathway from the precursor to the generation of lysine in Agaricomycetes [10] (Figure 1). These intermediates and enzymes are not only an important basis for exploring lysine biosynthesis but are also significant targets for the development of antifungal drugs [11,12].

The origin and evolution of the lysine biosynthetic pathway is a key step in cellular evolution because it changes the dependence of primitive cells on the external environment [9]. Based on the conservation of the amino acid sequences of specific enzymes in the pathways, Velasco and colleagues showed that the enzymes in the DAP pathway are related to arginine metabolism, and the enzymes in the AAA pathway are involved in leucine metabolism [13]. This is the first simple description of the evolutionary relationships of the lysine biosynthetic pathway, but this study does not cover the relationship between the lysine biosynthesis pathway and the evolution of species in Basidiomycota [13]. Torruella et al. explained the evolutionary history of lysine in eukaryotes based on the phylogenetic relationship between α-aminoadipate reductase (AAR) and LysA genes, and confirmed that the AAR gene is a molecular synapomorphy of fungi and protist Corallochytrium limacisporum [14]. However, the study only involves the homology of AAR from four species in Basidiomycota. Among the eight enzymes in the AAA pathway, homocitrate synthase (HCS) catalyzes the first step in lysine biosynthesis. The condensation of acetyl-CoA and α-ketoglutarate to form homocitrate is considered to be the rate-limiting step in the biosynthesis of lysine [2]. AAR catalyzes the reduction of α-aminoadipate to a semialdehyde, which is the most striking transformation in the biosynthesis of lysine in fungi. It is considered to be a completely unique mechanism in the primary metabolism of fungi [10]. The last step of the AAA pathway of lysine biosynthesis is a key step catalyzed by saccharopine dehydrogenase (SDH) in higher fungi. SDH catalyzes the cleavage of saccharopine with the aid of NAD+ to generate α-ketoglutarate and L-lysine. [10]. In recent years, the number of Agaricomycetes species whose genomes and transcriptomes have been sequenced is increasing, providing us with indispensable sequences with which to study the evolution of lysine biosynthesis in Agaricomycetes [15,16].

To date, the evolutionary relationship of lysine biosynthesis in Agaricomycetes is unclear. In this study, the phylogenetic relationship of lysine biosynthesis in Agaricomycetes was analyzed based on the reported sequences of the enzymes involved in the AAA pathway, especially HCS, AAR, and SDH, and the structural characteristic of proteins and the binding sites in predicted spatial structures. There is evolutionary conservation of lysine biosynthesis with evolution at the order level in Agaricomycetes.

## 2. Materials and Methods

### 2.1. Differential Expression Analysis of Genes by RNA-seq

The expression values of the eight genes of the enzymes in the AAA pathway of Coprinopsis cinerea #326 (GEO accession: GSE125184) and Lentinus tigrinus RLP-9953 (GEO accession: GSE125190) at different developmental stages by RNA-seq were downloaded from the GEO database in NCBI (https://www.ncbi.nlm.nih.gov/geo/ (accessed on 12 December 2021)). The transcriptome data were obtained from JGI database (https://jgi.doe.gov/ (accessed on 12 December 2021)) [16]. GraphPad 8.01 software was used for statistical analysis of expression level of genes.

### 2.2. Collection and Assembly of Sequences

The amino acid sequences of eight enzymes in the AAA pathway were downloaded from the NCBI database (www.ncbi.nim.nih.gov (accessed on 9 October 2021)). BLASTP analysis was done with the reported amino acid sequences of HCS (AGG53996.1) from F. velutipes, AAR (XP_001879618.1) from Laccaria bicolor, and SDH (QCX08356.1) from F. velutipes, and the homologous amino acid sequences from Agaricomycetes and Tremellomycetes were downloaded from the NCBI database for phylogenetic analysis [1,14,17]. This sequence information is listed in Tables S1–S3. The nucleotide sequences of six genes, including LSU, SSU, ITS (5.8S), RPB1, RPB2, and EF1-α, were downloaded and used to construct the phylogenetic tree of species (Table S4) [18,19].

### 2.3. Phylogenetic Analysis of Species and Enzymes Involved in the AAA Pathway

Sequence Matrix 1.7.8 was used to splice the above combination of six genes to construct the phylogenetic tree of the species. The amino acid sequences of HCS, AAR, SDH, HAH, HIDH, AAT, and SDR were aligned by MEGA 6.06 software, and then were manually adjusted. Phylogenetic trees of HCS, AAR, SDH, HAH, HIDH, AAT, and SDR were constructed by RAxML 1.3.1 software. The default PROTGAMMA (DAYHOFF) was selected for the amino acid replacement model and the maximum likelihood (ML) method was used for clustering. The nucleotide substitution model selected the default GTRGAMMA, and clustering was performed by the ML method with other parameters identical to the above constructions [18]. The strains of Tremellomycetes were regarded as the outgroup, and the reliability of internal branch was evaluated with 1000 bootstrap resampling. The phylogram was viewed and modified with FigTree (version 1.4.4).

### 2.4. Conservative Analysis of the Primary and Secondary Structures of HCS, AAR, and SDH

The conserved domains in the primary structures of HCS, AAR, and SDH in each species were analyzed by NCBI’s Conserved Domain Database (http://www.ncbi.nlm.nih.gov/Structure/cdd/wrpsb.cgi (accessed on 9 October 2021). The secondary structures analysis of the enzymes were predicted with SOPMA (https://npsa-prabi.ibcp.fr/cgi-bin/npsa_automat.pl?page=npsa_sopma.html (accessed on 9 October 2021) [20].

### 2.5. 3-D Structure Predictions of HCS, AAR, and SDH

The amino acid sequences of HCS (AGG53996.1), AAR (XP_001879618.1), and SDH (QCX08356.1) were submitted to I-TASSER webserver (https://zhanggroup.org/I-TASSER/ (accessed on 18 November 2021)) to predict the three-dimensional (3-D) structures [21,22]. The 3-D structures of these proteins were visualized in the Pymol 2.5 software package. All of the parameters were default.

### 2.6. Conservative Motif Analysis of Binding Sites in 3-D Structures of HCS, AAR, and SDH

The binding sites of substrate or cofactor in the 3-D structures of HCS, AAR, and SDH were then attained by sequence alignment based on the binding sites of the known homologous proteins in the PDB database. The conservative motifs analysis of the substrates or cofactor binding sites in homologous sequences of the three proteins were queried in MEME 5.4.1 (Multiple EM for Motif Elicitation, https://meme-suite.org/meme/tools/meme (accessed on 18 November 2021)) [23]. The search parameters were as follows: number of different motifs with 20, minimum motif width with 6, maximum motif width with 16, and other parameters were default.

## 3. Results

### 3.1. RNA-seq Analysis of the Genes of the Eight Enzymes Involved in the AAA Pathway

RNA-seq analysis showed significant differences in the expression levels of genes of the eight enzymes in the AAA pathway at different development stages and in different species [16]. The expression of genes in C. cinerea and L. tigrinus is shown in Figure S1. The results showed that HCS is almost at a higher level of expression at all of the development stages in the two strains, the expression levels of genes of HCS, AAT, AAR, and SDH are at a higher level in C. cinerea, and the expression levels of genes of HCS, HAH, HIDT, AAR, SDR, and SDH are at a higher level in L. tigrinus. Genes expression at the transcriptional level reflected the production of lysine at the different development stages. There was a correlation between Fvhcs gene expression and lysine production in the different developmental stages of F. velutipes based on the transcripmental analysis [1]. Our previous study also shows that the field of lysine achieves the high peak of production at the earlier stage in fermentation [24].

### 3.2. Phylogenetic Analysis of HCS in Agaricomycetes

**Figure 2:**
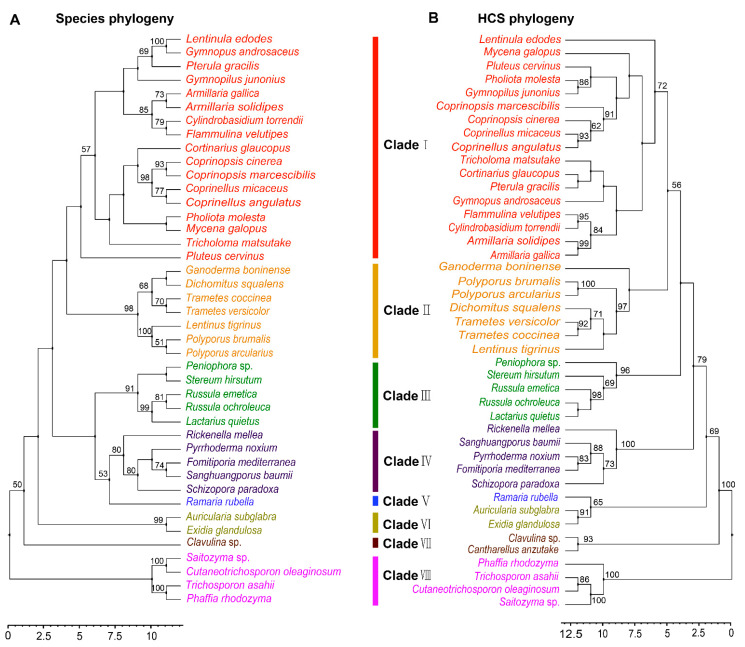
Phylogenetic relationship of both species and HCS homologous sequences in Agaricomycetes. (**A**) Maximum likelihood tree showing the relationships among different orders of Agaricomycetes based on LSU, SSU, RPB1, RPB2, ITS (5.8S), and EF1-α genes with species in Tremellomycetes as the outgroup. (**B**) Maximum likelihood tree showing the relationships among different orders of Agaricomycetes based on HCS amino acid sequences with species in Tremellomycetes as the outgroup. MP bootstrap values (≥50%) of each clade are indicated at nodes. Scale bar in the upper left indicates substitutions per site. (Clade I: Agaricales, Clade II: Polyporales, Clade III: Russulales, Clade IV: Hymenochaetales, Clade V: Gomphales, Clade VI: Auriculariales, Clade VII: Cantharellals, and Clade VIII: Tremellomycetes).

Phylogenetic analysis of FvHCS in F. velutipes and other homocitrate synthase proteins from diverse fungal species in Agaricomycotina is congruent with the current fungal systematics, and these orthologous sequences may be used as DNA bar codes to assist in classifying fungi relationships [1]. To examine the molecular evolutionary mechanism of lysine biosynthesis in Agaricomycetes, we firstly performed a phylogenetic analysis of HCS, and then compared the phylogenetic relationships of species in Agaricomycetes. The 43 species having orthologous HCS sequences were distributed in 19 families and seven orders in Agaricomycetes (Table S1). These strains belong to the Agaricales, Polyporals, Russulales, Hymenochaetales, Auriculariales, Gomphales, and Cantharellales, and have a robust phylogenetic framework at the order level. However, there was some confusion at the family level, including species in families of Omphalotaceae and Pluteaceae. Horizontal gene transfer (HGT) could occur among multiple species at the family level during the long evolutionary process. The results showed the phylogenetic relationship between species and HCS at the order level (Figure 2A,B). This indicated that the evolution of HCS was consistent with the evolution of species at the order level, but there were some differences at the family level in Agaricomycetes.

### 3.3. Phylogenetic Analysis of AAR in Agaricomycetes

**Figure 3:**
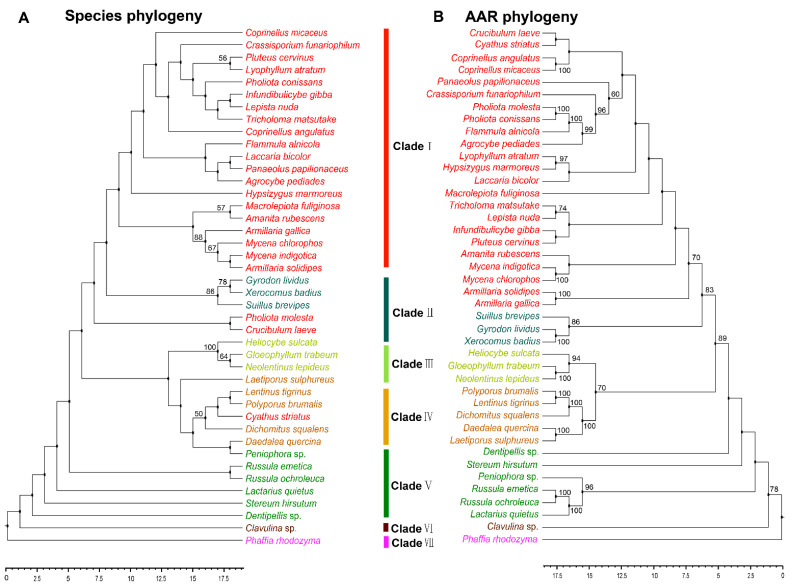
Phylogenetic relationship both species and AAR homologous sequences in Agaricomycetes. (**A**) Maximum likelihood tree showing the relationships among different orders of Agaricomycetes based on LSU, SSU, RPB1, RPB2, ITS (5.8 S), and EF1-α genes with species in Tremellomycetes as the outgroup. (**B**) Maximum likelihood tree showing the relationships among different orders of Agaricomycetes based on AAR amino acid sequence with species in Tremellomycetes as outgroup. MP bootstrap values (≥50%) of each clade are indicated at nodes. Scale bar in the upper left indicates substitutions per site (Clade I: Agaricales, Clade II: Boletales, Clade III: Gloeophyllales, Clade IV: Polyporales, Clade V: Russulales, Clade VI: Cantharellals, and Clade VII: Tremellomycetes).

The reaction catalyzed by AAR is arguably the key step in the fungal lysine biosynthesis pathway, and is an essential mechanism in primary metabolism [10,25]. AAR is regarded as a key enzyme in the evolution of fungal lysine synthase [26]. Analyzing the phylogeny of AAR can help to understand how lysine biosynthesis evolved in fungi. The AAR homologous sequences of 41 strains in Agaricomycetes with sequences of outgroup species in Tremellomycetes were used. These 41 species with reported AAR belonged to 25 families and six orders in Agaricomycetes (Table S2). However, not all of the homologous sequences of AAR in the strains having HCS sequence were obtained from the NCBI database. There may be two reasons for it. On the one hand, there is a branch pathway in the AAA pathway of lysine synthesis which is located upstream of the AAR enzyme. The five enzymes LysX, LysZ, LysY, LysJ, and LysK sequentially catalyze the conversion of α-aminoadipate to lysine. This branch pathway typically exists in bacteria and archaea [10]. On the other hand, some enzymes with highly homologous sequences may be involved in the synthesis of other molecules. With the exception of Pholiota molesta, Crucibulum laeve and Cyathus striatus, other species have a robust phylogenetic framework at the order level. The evolutionary types of the phylogeny of AAR with Agaricales, Polyporales, Russulales, and Cantharellales were consistent with those of HCS. The phylogenetic tree shows the phylogenetic relationship between species and AAR at the order level in Agaricomycetes (Figure 3A,B). The results indicated that the evolution of AAR was consistent with the evolution of species at the order level.

### 3.4. Phylogenetic Analysis of SDH in Agaricomycetes

**Figure 4:**
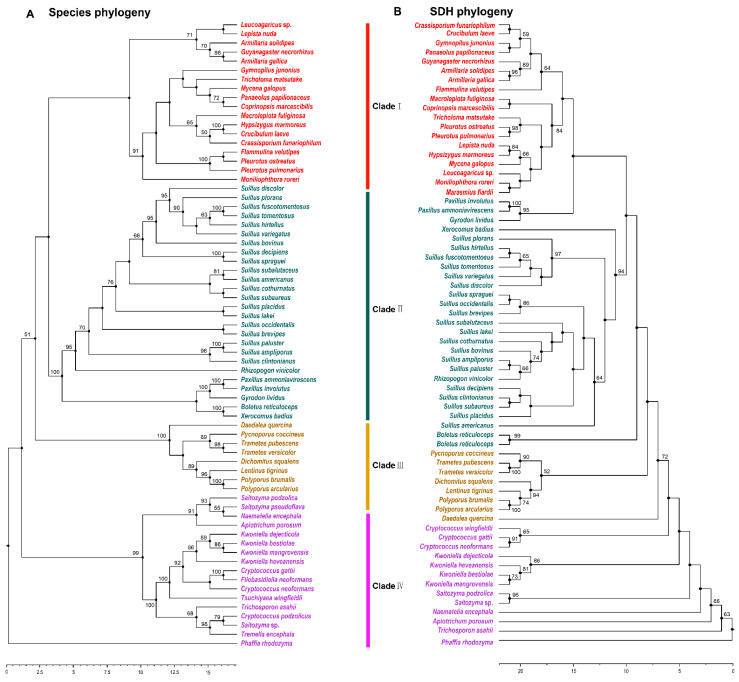
Phylogenetic relationship both species and SDH homologous sequences in Agaricomycetes. (**A**) Maximum likelihood tree showing the relationships among different orders of Agaricomycetes based on LSU, SSU, RPB1, RPB2, ITS (5.8 S), and EF1-α genes with species in Tremellomycetes as the outgroup. (**B**) Maximum likelihood tree showing the relationships among different orders of Agaricomycetes based on SDH amino acid sequence with species in Tremellomycetes as the outgroup. MP bootstrap values (≥50%) of each clade are indicated at nodes. Scale bar in the upper left indicates substitutions per site (Clade I: Agaricales, Clade II: Boletales, Clade III: Polyporales, and Clade IV: Tremellomycetes).

SDH catalyzes the final step of lysine biosynthesis in the AAA pathway [10]. Overexpression of *Fvsdh* can improve lysine biosynthesis in *F. velutipes* [27]. The phylogenetic analysis of SDH indicated that 53 species with outgroup species in Tremellomycetes were members of 22 families and three orders in Agaricomycetes. The majority of the species containing SDH in Agaricomycetes existed in Agaricales and Boletales, and had a robust phylogenetic framework at the order level. The phylogenetic structure was disorganized at the family level (Figure 4A,B). This result was consistent with the results of a previous analysis of HCS and AAR.

### 3.5. Phylogenetic Analysis of Other Enzymes in the AAA Pathway

In addition to the previously analyzed enzymes, the AAA pathway includes another five enzymes—HCD, HAH, HIDH, AAT, and SDR [2,10] (Figure 1). Since the function of HCD was confirmed in 1964, there have been only a few homologous sequences of Agaricomycetes in the NCBI database, including homologous sequences of HCD from *Grifola frondose* (OBZ73766.1), *Trametes pubescens* (OJT09835.1), *Hypsizygus marmoreus* (RDB23442.1) and *Sparassis crispa* (XP_027609096.1). Thus, it is difficult to probe the conservation and molecular evolution of Agaricomycetes. Eight homologous sequences of AAT were identified in four species in Agaricomycetes in the NCBI database that are members of MocR family based on a BLASTP analysis of AAT (XP_002910512.1) in C. cinerea [28]. Phylogenetic analysis of the homology of AAT showed that they are clustered into two different branches, namely PLP-dependent transferase and TdiD protein (Figure S3). They were predicted to be kynurenine/alpha-aminoadipate aminotransferase and to be involved in transcription and amino acid transport. They have the same conserved domains of AAT and can catalyze the reversible exchange of the amino group on one molecule and the ketone group on the other molecule, which suggests that they could have descended from a common ancestor. Moreover, BLASTP analysis of these three enzymes in the AAA pathway was conducted based on the sequences of HAH (PBL03870.1) in *Armillaria gallica* [29], HIDH (XP_001836919.1) in *C. cinerea* [28], and SDR (KIY66865.1) in *Cylindrobasidium torrendii* [30]. There are 72 homologous sequences of HAH, 86 homologous sequences of HIDH, and 31 homologous sequences of SDR in the NCBI database. The phylogenetic analysis of HCS, AAR, and SDH also showed that all of them are more highly conserved at the order level in Agaricomycetes (Figures S2, S3, and S5).

### 3.6. Conservation of Domains in the Primary Structures of HCS, AAR, and SDH

**Figure 5:**
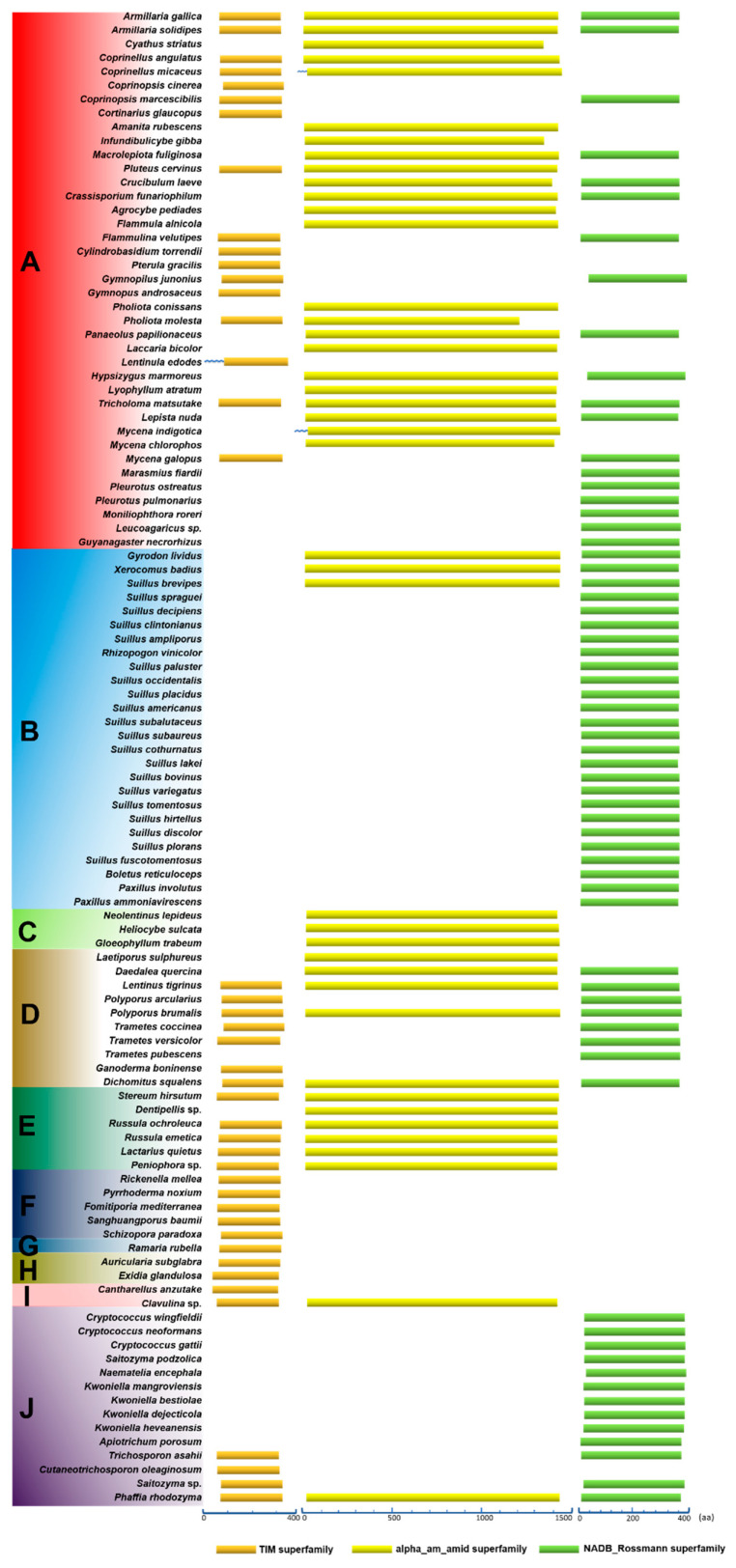
Conserved domains in the primary structures of HCS, AAR, and SDH in Agaricomycetes. (**A**) Agaricales, (**B**) Boletales, (**C**) Gloeophyllales, (**D**) Polyporales, (**E**) Russulales, (**F**) Hymenochaetales, (**G**) Gomphales, (**H**) Auriculariales, (**I**) Cantharellals, and (**J**) Tremellomycetes. Abbreviations: “TIM superfamily”: These members share a conserved triose-phosphate isomerase (TIM) barrel domain consisting of a core beta(8)-alpha (8) motif with the eight parallel beta strands forming an enclosed barrel surrounded by eight alpha helices. The domain has a catalytic center containing a divalent cation-binding site formed by a cluster of invariant residues that cap the core of the barrel; “alpha_am_amid superfamily”: Members of this protein family are L-aminoadipate-semialdehyde dehydrogenase (EC 1.2.1.31). It is also called alpha-aminoadipate reductase. Lysine is synthesized via aminoadipate in higher fungi; “NADB Rossmann superfamily”: Lysine-ketoglutarate reductase/saccharopine dehydrogenase. “**﹏**”: It represents at least 331 amino acids.

The conservation of amino acid sequences of proteins is affected by their restriction of molecular function and by molecular evolution. Typically, one protein is composed of several different domains with varying evolutionary origins and functions. The functional characteristic conservative domains of HCS, AAR, and SDH were analyzed using the NCBI Conserved Domain Database (Figure 5). Firstly, 43 HCS homologous sequences were analyzed in Agaricomycetes. Although the composition of amino acid sequences was distinctly different in HCS, AAR, and SDH. Their common features indicated that they are members of the DRE_TIM_HCS metallolyase superfamily. Since members of this family have similar structural features of the proteins, they can perform similar functions, such as catalyzing the condensation of acetyl-CoA with α-ketoglutarate to form homocitrate. There is a motif syntapomorphic to the Agaricomycotina, which revealed the conserved nature of HCS that could aid in identifing homocitrate synthase in other species of Agaricomycotina [1]. Our data indicated that HCS has been highly conserved, which is conducive to the evolution of a more perfect pathway for lysine synthesis in higher fungi (Table S1).

Secondly, 42 homologous sequences of AAR in strains of Agaricomycetes were analyzed. The amino acid sequences of AAR were significantly longer than the amino acid sequences of HCS, and they were all members of the alpha_am_amid superfamily (Figure 5). The results indicate that the characteristics of domains in the primary structures of AAR are also highly conserved (Table S2). Alternatively, the amino acid sequences of SDH from 66 species in Agaricomycetes and Tremellomycetes were analyzed. The amino acid sequences of SDH with approximately 0 and 400 residues were predicted to be part of the NADB_Rossman superfamily. Only SDHs in the NADB_Rossman superfamily can catalyze the cleavage of saccharine to produce lysine and α-ketoglutarate. Therefore, we compared the members of the NADB_Rossman in the 66 strains. The results showed that the amino acid sequences of SDHs are highly conserved and do not change significantly owing to the differences in the species (Table S3). The domains in the primary structures of HCS, AAR, and SDH provide additional evidence that the AAA pathway of lysine biosynthesis was highly conserved in Agaricomycetes.

### 3.7. High Conservation of the Secondary Structures of HCS, AAR, and SDH

The secondary structure of protein is primarily composed of alpha helices, extended strands, beta turns, and random coils. Based on the homologous sequences of HCS, AAR, and SDH in Agaricomycetes species, characteristics of the secondary structure of these homologous sequences were analyzed (Table S5). Alpha helices and random coils are the dominant forms in the secondary structures of HCS, AAR, and SDH. The ranges of variation of the alpha helices of proteins in different species were 8.20%, 3.99%, and 5.53%, respectively. For the extended strand, the ranges of variation of HCS, AAR, and SDH in different species were 3.50%, 2.17%, and 3.18%, respectively. The ranges of variation of the beta turns of HCS, AAR, and SDH in different species were 2.49%, 1.58%, and 2.91%, respectively. Finally, for the random coil, the ranges of variation of HCS, AAR, and SDH in different species were 8.46%, 1.91%, and 6.79%, respectively (Figures S6–S8). These results indicated that the secondary structures of HCS, AAR, and SDH do not differ conspicuously in different species. In addition, the main reason for these subtle differences could be that there is not a remarkable difference at the boundary of secondary structures. The large number of alpha helices and random coils lays the foundation for maintenance of the stability and complexity of the protein conformation. The characteristics of secondary structures of HCS, AAR, and SDH have a high degree of similarity and conservation. HCS, AAR, and SDH are derived from their own ancestors in different species. Thus, lysine biosynthesis is inseparable with the evolution of HCS, AAR, and SDH. The evolutionary conservation of HCS, AAR, and SDH at the secondary structures also further reflects the evolutionary trend of lysine biosynthesis in Agaricomycetes.

### 3.8. 3-D Structures of HCS, AAR, and SDH and the High Conservation of Binding Sites

**Figure 6:**
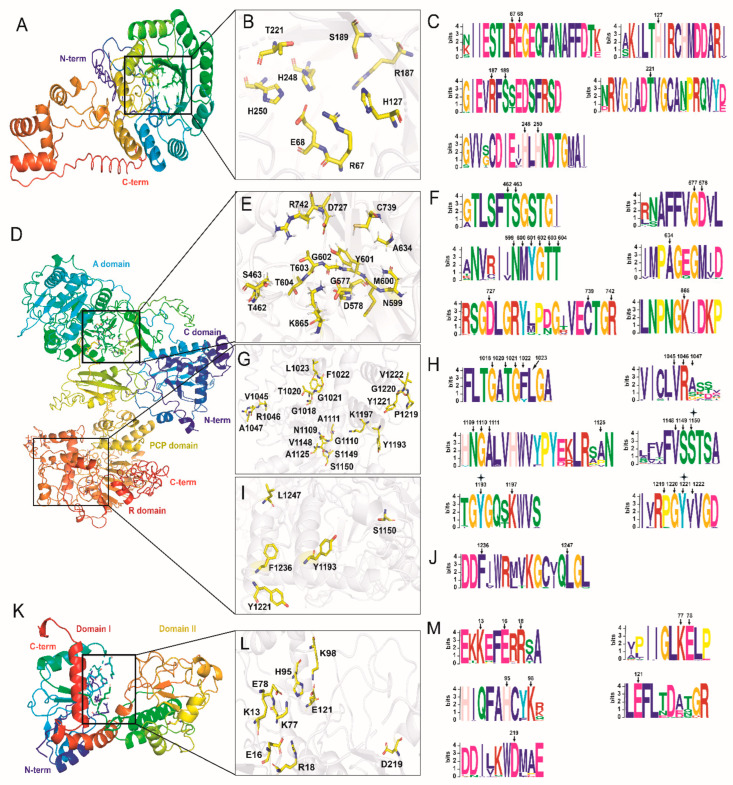
Prediction of 3-D structures and interdomain interactions of substrate molecules trapped in enzymatic progress of HCS, AAR, and SDH. (**A**) 3-D structure of FvHCS in *F. velutipes*, (**B**) interactional binding sites of substrate molecule in HCS, (**C**) conservative motif of interactional binding sites in homologous sequences of HCS by MEME, (**D**) 3-D structure of LbAAR in L. bicolor, (**E**) interactional binding sites of AMP molecule in AAR, (**F**) conservative motif of interactional binding sites of AMP in homologous sequences of AAR by MEME, (**G**) interactional binding sites of NAD(**P**) in AAR, (**H**) conservative motif of interactional binding sites of NAD(P) in homologous sequences of AAR by MEME, (**I**) interactional binding sites of putative substrate molecule in AAR, (**J**) conservative motif of interactional binding sites of putative substrate molecule in homologous sequences of AAR by MEME, (**K**) 3-D structure of FvSDH in *F. velutipes*, (**L**) interactional binding sites of substrate saccharopine in SDH, and (**M**) conservative motif of interactional binding sites in homologous sequences of SDH by MEME. Abbreviations: “N-term”: N-terminal, the starting site for protein synthesis. “C-term”: C-terminal, the termination site of protein synthesis. “C domain”: Condensation domain in NRPS. “A domain”: Adenylation domain in NRPS. “PCP domain”: Peptidyl carrier protein in NRPS. “R domain”: extended (e) SDRs domain in NRPS. “Domain I”: N-terminal domain in SDH, substrate-bound domains. “Domain II”: C-terminal domain in SDH, NAD bound domain.

The three-dimensional (3-D) structure of FvHCS in F. velutipes was predicted by I-TASSER (Figure 6A). The result showed that FvHCS is composed of both an N-terminal catalytic domain and a C-terminal hybrid domain, in which the C-score was −1.41 Å. The TM-score was 0.54 ± 0.15 Å, and the RMSD was 10.4 ± 4.6 Å. These data truly reflected the tertiary structure of FvHCS. Ten structural analogues of FvHCS were identified by conducting comparisons in the PDB database, including with members of the archaea (6ktqA), bacteria (6e1jA, 3figB, 4ov4A, 3bliA, 3a9iA, 3rmjA and 6ndsA), fungi (3ivtB), and Metazoa (2cw6A) (Table S7). The homology between FvHCS and 3ivtB in *Schizosaccharomyces pombe* was 69.4%. The results indicated that HCS is widely distributed in different species and extremely highly conserved in fungi. We deduced the substrate-binding sites of FvHCS based on the catalytic function of 3ivtB (Figure 6B) [31]. The substrate-binding pocket of FvHCS is in the classical (α/β) 8 TIM barrel, and the substrate molecule is fixed in the center of the β-barrel through hydrogen bonds and salt bridges that interact with the surrounding amino acid residues. The amino acid residues of Glu-68, His-248, and His-250 interacted with the substrate by binding divalent Zn^2+^, and Arg-67,Thr-221, His-127, Arg-187, and Ser-189 which bind to the specific groups in the substrate through hydrogen bonding and catalyze the condensation of α-ketoglutarate with acetyl-CoA to form homocitrate (Figure 6B). The conservative motifs of substrate-binding sites of HCS were identified in 30 species in Agaricomycetes by MEME. There were eight amino acid residues highly conserved and widely distributed in the orders of Agaricales, Polyporales, Russulales, Hymenochaetales, Gomphales, Auriculariales, Cantharellales, Trichosporonales, and Cystofilobasidiales (Figure 6C).

The 3-D structure of LbAAR with 1420 amino acid residues in *L. bicolor* was predicted by I-TASSER. It consists of four domains, including the condensation (C) domain (residues 10–116), adenylation (A) domain (residues 288–862), peptidyl carrier protein (PCP) (residues 885–951), and extended (e) SDRs (residues 1013–1317) (Figure 6D). LbAAR was predicted to be a non-ribosomal peptide synthetase (NRPS), in which the C-score was −1.61 Å, the TM-score was 0.52 ± 0.15 Å, and the RMSD was 13.8 ± 3.9 Å. NRPS is generally considered to be one type of enzyme in secondary metabolic synthesis, and it is also indispensable in lysine synthesis. The reaction catalyzed by AAR is thought to be a unique reaction mechanism that involves both adenylation and reduction in primary metabolism [10]. Ten structural analogues of LbAAR were identified by comparison in PDB database, including bacteria (4zxhA, 6mfzA, 2vsqA, 6n8eA, 4zxjA, 5u89A, 1amuA, and 1mdbA), fungi (1ry2A) and metazoa (2d1rA) with relatively low homology (Table S7). 1ry2A in Saccharomyces cerevisiae was identified as acetyl-coenzyme A synthetase. The 3–D structure of 1ry2A was only similar to the A domain in LbAAR and had no connection with other domains [32]. The catalytic reaction by LbAAR is a three-step process from α-aminoadipate to semialdehyde. ATP and NAD(P)H play roles in the first step of adenylation and the second step of reduction, respectively, and there are no cofactors involved in the third step [2]. Based on the catalytic function of AAR in bacteria, the binding sites of cofactors and substrates in LbAAR were deduced (Figure 6E,G,I) [33]. There is one bigger N-terminal domain and one smaller C-terminal domain in the A domain, and AMP is combined on the interfaces of these two domains. The binding sites in A domain consist of 15 amino acid residues, including Thr-462, Ser-463, Gly-577, Asp-578, Asn-599, Met-600, Tyr-601, Gly-602, Thr-603, Thr-604, Ala-634, Asp-727, Cys-739, Arg-742, and Lys-856 (Figure 6E). The conservative motifs of binding sites of AMP in AAR were identified in 27 species in Agaricomycetes by MEME. There were 15 amino acid residues highly conserved and widely distributed in the orders of Agaricales, Boletales, Gloeophyllales, Polyporales, Russulales, and Cantharellales (Figure 6F). The core structure of extended (e) SDRs contains the conserved Rossmann-fold structure with a TGXXGXXG cofactor binding motif (TGATGFLG) and a YXXXK active site motif (YGQTK). There were 21 amino acid residues of binding sites with NAD(P) as cofactor in LbAAR, and, in 27 species, all of these amino acid residues were conserved except for Phe-1022, Ala-1047, Ala-1125, Pro-1219, and Val-1222 (Figure 6H). The amino acid residues as binding sites of substrates in extended (e) SDRs were predicted to contain Ser-1150, Tyr-1193, Tyr-1221, Phe-1236, and Leu-1247 (Figure 6I). These five amino acid residues are high conserved in Agaricomycetes (Figure 6J). Alternatively, the three amino acid residues of Ser-1150, Tyr-1193, and Tyr-1221 are involved in binding sites in both NAD(P) and substrate molecules. Moreover, the Ser residue in the PCP domain is usually an important active site to load aminoyl-AMP. Ser-915 in the PCP domain of LbAAR is hypothesized to exercise this function, and it is also conserved in Agaricomycetes (Figure S9). The functional analysis of these amino acid residues as binding sites in NRPS reveals the conservative property of AAR in the reduction of α-aminoadipate.

The 3-D structure of FvSDH with 368 amino acid residues in F. velutipes was pre-dicted by I-TASSER. FvSDH consists of two domains with similar size, namely an N-terminal domain (Domain I, 4–136 residues, 331–368 residues) and a C-terminal domain (Domain II, 137–330 residues) (Figure 6K). Domain I has the main binding sites of the substrate saccharopine. It contains one α/β fold and is similar to the topology of dinucleotide binding domains [34]. Domain II has the main binding sites of cofactor NAD, and it contains one typical Rossmann fold (α-β-α-β-α-β). The substrate primarily binds in the crack between the two domains, and the enzyme has broad substrate specificity [35]. Ten structural analogues of FvSDH were identified by comparison in the PDB database, including fungi (2qrlA), bacteria (1pjcA, 1f8gA, 1m2wA, and 1gcaA), metazoa (1gz3A, 1p0fA, 1hrkA, 1pl6A), and viridiplantae (1u1uA) (Table S7). The homology between FvSDH and 2qrlA in *S. cerevisiae* was 54.3%, which showed that the SDH analogues are widely distributed and highly conserved in fungal species. Based on the catalytic function of SDH in S. cerevisiae, the substrate-binding sites of FvSDH were predicated (Figure 6L). The substrate-binding sites of FvSDH are in the interface of domain I. Arg-18 interacts with the two carboxyl groups of saccharopine, and Glu-121 forms hydrogen bonds with the imine part of saccharopine. Lys-98 interacts with the amino acid tail of saccharopine at the edge of the binding site of substrate. Both side chains of Lys-77 and His-95 are suitable as acid−base catalysts in the reaction [36]. The Asp-219 residue is not a binding site of the substrate, but it could play a key role in binding the ribose portion of the AMP molecule (Figure 6M). These key substrate binding sites are highly conserved in Agaricomycetes, which suggests that the catalytic function of SDHs on substrate saccharopine is also highly conserved in functional evolution.

## 4. Discussion

Lysine is one of the essential amino acids for human growth and development, and it is prevalent in mushrooms of Agaricomycetes [37]. Our understanding of lysine biosynthesis at the molecular level has enormously in-creased with the growth of genomic information [1,27]. Therefore, phylogeny of the ly-sine biosynthetic pathway will help to reveal the evolutionary course of lysine biosyn-thesis. The AAA pathway is responsible for the production of lysine in Agaricomycetes species [1,27,38]. Eight enzymes involved in AAA pathway, including HCS, HCD, HAH, HIDH, AAT, AAR, SDR, and SDH, are primarily involved in the biosynthesis of lysine in higher fungi [10,39]. In addition, the phylogenetic relationship of the three enzymes of HCS, AAR and SDH in the AAA pathway is consistent with the evolution-ary relationship of species in Agaricomycetes [18,40]. The homologous sequences of HCS, AAR, and SDH are known only in 70 species of 10 orders in Agaricomycetes, which confirms the widespread existence of lysine in Agaricomycetes, and further up-dates the number of lysine-producing species in Agaricomycetes [13]. Owing to the limited resources of strains contained known sequences of HCS, AAR and SDH, we have not identified the existence of these three enzymes in other orders of Agaricomy-cetes. Even so, our results could only reflect the phylogenetic relationships of the mi-nority of strains reported in Agaricomycetes. It provides concepts to reveal the phylo-genetic relationships of lysine biosynthesis in Basidiomycota fungi in more detail. The phylogenetic relationships of other enzymes in AAA pathway were analyzed in Agaricomycetes based on the known enzymes of homologous sequences. The results showed that the enzymes involved in AAA pathway of lysine biosynthesis are more higer conserved at the order level in Agaricomycetes in addition to HCD and AAT. There are no adequate numbers of homologous sequences of them to construct a phylogenetic analysis.

The phylogenetic relationships of HCS, AAR, and SDH are similar at the order level in Agaricomycetes. But the species with each enzyme used in the phylogenetic tree had very obvious differences. In all of the phylogenetic trees, there were only eight species, including *A. gallica*, *A. solidipes*, *Coprinellus angulatus*, *L. tigrinus*, *Polyporus brumalis*, *Dichomitus squalens*, *Russula ochroleuca*, and R. emetic, and there were only one or two known enzymes in other species. There may be three possible reasons for this: (1) the homologous enzymes in other species have not been identified; (2) the enzymes in other species have evolved to have an unrecognizable primary structure; or (3) the enzymes that are lacking in other species may be supplemented by enzymes with low specificity in other metabolic pathways [39,41]. Moreover, the evolutionary relationship between *Pholiota molesta*, *C. laeve*, and *C. striatus* was chaotic in the phylogeny of AAR, and it was not consistent with the evolutionary relationship of HCS and SDH. We hypothesized that the AAR from these three species may be undergone horizontal gene transfer from other species during the evolution process [39].

The 3-D structure of proteins largely determine the biological functions of enzymes, and the binding sites of substrate are the important factor in studying the functional evolution of proteins. Therefore, understanding of the 3-D structure of proteins and the conservation of binding sites is highly significant for revealing the functional evolution of enzymes. Crystal structures of HCS and SDH in yeast provide the basis for predicting their 3-D structures in fungi [31,34]. There was a high degree of homology of the 3-D structures between HCS and SDH in *F. velutipes* and 3ivtB in *S. pombe* and 2qrlA in *S. cerevisiae*. The results revealed the high degree of conservation of 3-D structures between HCS and SDH in Agaricomycetes, even in fungi. Moreover, our sequence alignment revealed that the binding sites in HCS and SDH are highly conserved in Agaricomycetes. The 3-D structural analysis of FvAAR in *F. velutipes* revealed that it is a member of NRPS, which catalyzes one unique reaction mechanism in the primary metabolism in fungi. The results of our prediction showed that the binding sites with AMP, NAD(P), and the substrate are highly conserved expect for five binding sites (Phe-1022, Ala-1047, Ala-1125, Pro-1219, and Val-1222) with NAD(P) in Agaricomycetes. There is no crystal structure of AAR homology of the enzymes to predict the AAR in fungi. The lower degree of homology between FvAAR and other homologous sequences of AAR in the PDB database could be the primary cause of the poor conservation in NAD(P) binding sites. We retrieved the 3-D structure of AAR (Login number: P07702) in *S. cerevisiae* S288c predicted by AlphaFold in UniProt database (https://www.uniprot.org/ (accessed on 18 November 2021)). Thus, it is necessary to study the complex model of interactions between LbAAR and substrates or cofactors through AlphaFold and molecular docking to reveal the molecular mechanism of the evolution of lysine biosynthesis. Furthermore, there is one conservative Ser site in the PCP domain of AAR in both *S. cerevisiae* and Agaricomycetes species [42]. The Ser site is an important target to study the interactions between NRPS and small molecules. The conservation of this Ser site provides additional evidence that reveals the preservation of the catalytic mechanism of AAR in fungi.

## 5. Conclusions

In this work, we revealed the phylogenetic relationship of lysine biosynthesis in Agaricomycetes with the enzymes involved in the AAA pathway, particularly HCS, AAR, and SDH. The results indicate that the evolution of lysine biosynthesis evolved in parallel with the evolution of species at the order level in Agaricomycetes. HCS, AAR, and SDH are highly conserved in their primary, secondary, tertiary structures, and the binding sites of substrates. The degree of structure homology of enzymes reflects the degree of conservation of the lysine biosynthesis pathway in Agaricomycetes during the evolutionary process. Based on analysis of 3-D structure of the three enzymes of HCS, AAR, and SDH in the AAA pathway and the conservation of catalytic residues of the binding sites, the catalytic function of these enzymes was highly conserved in evolution in Agaricomycetes. Lysine biosynthesis could have served as an evolutionary precursor to more biosynthesis of other essential amino acids.

## Data Availability

The data presented in this study are available in this manuscript, and constructs can be requested from the corresponding author.

## Supplementary Materials

The following are available online at https://www.mdpi.com/article/10.3390/jof8010037/s1, Table S1: The homologous sequences of HCS used to construct the phylogenetic tree in this study. Table S2: The homologous sequences of AAR used to construct the phylogenetic tree in this study. Table S3: The homologous sequences of SDH used to construct the phylogenetic tree in this study. Table S4: The sequences used to construct the phylogenetic tree of species in this study. Table S5: Characteristics of the predicted secondary structures of HCS, AAR, and SDH in Agaricomycetes. Table S6: Sequences used for 3-D structure prediction and conservation analysis of binding sites of HCS, AAR, and SDH. Table S7: The proteins with similar 3-D structures to the reported proteins of HCS, AAR, and SDH in the PDB database. Figure S1: Expression levels of the genes of eight enzymes involved in the AAA pathway in different development stages by RNA-seq analysis in Coprinopsis cinerea and Lentinus tigrinus. Figure S2: Phylogenetic tree of HAH in Agaricomycetes with Tremellomycetes as outgroup. Figure S3: Phylogenetic tree of HIDH in Agaricomycetes with Tremellomycetes as outgroup. Figure S4: Phylogenetic tree of AAT in Agaricomycetes. Figure S5: Phylogenetic tree of SDR in Agaricomycetes with Tremellomycetes as outgroup. Figure S6: Characteristics of the secondary structure of HCS in Agaricomycetes. Figure S7: Characteristics of the secondary structure of AAR in Agaricomycetes. Figure S8: Characteristics of the secondary structure of SDH in Agaricomycetes. Figure S9: Multiple sequence alignment analysis of PCP domain in AAR from S. cerevisiae with PCP domains from 27 species in Agaricomycetes.

## References

[1] Liu F., Wang W., Chen B.Z., Xie B.G. (2015). Homocitrate synthase expression and lysine content in fruiting body of different developmental stages in *Flammulina velutipes*. Curr. Microbiol..

[2] Xu H., Andi B., Qian J., West A.H., Cook P.F. (2006). The α-aminoadipate pathway for lysine biosynthesis in fungi. Cell Biochem. Biophys..

[3] Yin J., Li Y.Y., Han H., Zheng J., Wang L.J., Ren W.K., Chen S., Wu F., Fang R.J., Huang X.G. (2017). Effects of lysine deficiency and Lys-Lys dipeptide on cellular apoptosis and amino acids metabolism. Mol. Nutr. Food Res..

[4] Astrom S., Vonderdecken A. (1983). Lysine deficiency reduces transcription activity and concentration of chromatin proteins reversibly in rat-liver. Acta Physiol. Scand..

[5] Beluhan S., Ranogajec A. (2011). Chemical composition and non-volatile components of Croatian wild edible mushrooms. Food Chem..

[6] Manninen H., Rotola-Pukkila M., Aisala H., Hopia A., Laaksonen T. (2018). Free amino acids and 5 ‘-nucleotides in Finnish forest mushrooms. Food Chem..

[7] Schmidt S., Sunyaev S., Bork P., Dandekar T. (2003). Metabolites: A helping hand for pathway evolution?. Trends Biochem. Sci..

[8] Cunchillos C., Lecointre G. (2007). Ordering events of biochemical evolution. Biochimie.

[9] Fondi M., Brilli M., Emiliani G., Paffetti D., Fani R. (2007). The primordial metabolism: An ancestral interconnection between leucine, arginine, and lysine biosynthesis. BMC Evol. Biol..

[10] Zabriskie T.M., Jackson M.D. (2000). Lysine biosynthesis and metabolism in fungi. Nat. Prod. Rep..

[11] Amich J., Bignell E. (2016). Amino acid biosynthetic routes as drug targets for pulmonary fungal pathogens: What is known and why do we need to know more?. Curr. Opin. Microbiol..

[12] Schobel F., Jacobsen I.D., Brock M. (2010). Evaluation of lysine biosynthesis as an antifungal drug target: Biochemical characterization of *Aspergillus fumigatus* homocitrate synthase and virulence studies. Eukaryot. Cell.

[13] Velasco A.M., Leguina J.I., Lazcano A. (2002). Molecular evolution of the lysine biosynthetic pathways. J. Mol. Evol..

[14] Miyauchi S., Kiss E., Kuo A., Drula E., Kohler A., Sanchez-Garcia M., Morin E., Andreopoulos B., Barry K.W., Bonito G. (2020). Large-scale genome sequencing of mycorrhizal fungi provides insights into the early evolution of symbiotic traits. Nat. Commun..

[15] Liu J., Wang J., Zhang D., Shang X., Tan Q. (2016). Analysis of genes related to lysine biosynthesis based on whole genome of *Flammulina velutipes*. Microbiol. China.

[16] Krizsan K., Almasi E., Merenyi Z., Sahu N., Viragh M., Koszo T., Mondo S., Kiss B., Balint B., Kues U. (2019). Transcriptomic atlas of mushroom development reveals conserved genes behind complex multicellularity in fungi. Proc. Natl. Acad. Sci. USA.

[17] Martin F., Aerts A., Ahren D., Brun A., Danchin E.G.J., Duchaussoy F., Gibon J., Kohler A., Lindquist E., Pereda V. (2008). The genome of *Laccaria bicolor* provides insights into mycorrhizal symbiosis. Nature.

[18] He M.-Q., Zhao R.-L., Hyde K.D., Begerow D., Kemler M., Yurkov A., McKenzie E.H.C., Raspé O., Kakishima M., Sánchez-Ramírez S. (2019). Notes, outline and divergence times of Basidiomycota. Fungal Divers..

[19] Li G.J., Zhao R.L., Zhang C.L., Lin F.C. (2019). A preliminary DNA barcode selection for the genus *Russula* (Russulales, Basidiomycota). Mycology.

[20] Geourjon C., Deleage G. (1995). SOPMA: Significant improvements in protein secondary structure prediction by consensus prediction from multiple alignments. Comput. Appl. Biosci..

[21] Yang J., Zhang Y. (2015). I-TASSER server: New development for protein structure and function predictions. Nucleic Acids Res..

[22] Zhang C., Freddolino P.L., Zhang Y. (2017). COFACTOR: Improved protein function prediction by combining structure, sequence and protein-protein interaction information. Nucleic Acids Res..

[23] Bailey T.L., Williams N., Misleh C., Li W.W. (2006). MEME: Discovering and analyzing DNA and protein sequence motifs. Nucleic Acids Res..

[24] Song Z., Zhao R., Zhang H., Wei P., Qi L., Chen G., Yin W.B., Li W. (2021). Rapid and accurate screening of lysine-producing edible mushrooms via the homocitrate synthase gene as a universal molecular marker. ACS Omega.

[25] Kalb D., Lackner G., Hoffmeister D. (2014). Functional and phylogenetic divergence of fungal adenylate-forming reductases. Appl. Environ. Microbiol..

[26] Nishida H., Nishiyama M. (2000). What is characteristic of fungal lysine synthesis through the alpha-aminoadipate pathway?. J. Mol. Evol..

[27] Liu J., Li Q., Jiang P., Xu Z., Zhang D., Zhang L., Zhang M., Yu H., Song C., Tan Q. (2019). Overexpression of the saccharopine dehydrogenase gene improves lysine biosynthesis in *Flammulina velutipes*. J. Basic Microbiol..

[28] Stajich J.E., Wilke S.K., Ahren D., Au C.H., Birren B.W., Borodovsky M., Burns C., Canback B., Casselton L.A., Cheng C.K. (2010). Insights into evolution of multicellular fungi from the assembled chromosomes of the mushroom *Coprinopsis cinerea* (*Coprinus cinereus*). Proc. Natl. Acad. Sci. USA.

[29] Sipos G., Prasanna A.N., Walter M.C., O’Connor E., Balint B., Krizsan K., Kiss B., Hess J., Varga T., Slot J. (2017). Genome expansion and lineage-specific genetic innovations in the forest pathogenic fungi *Armillaria*. Nat. Ecol. Evol..

[30] Floudas D., Held B.W., Riley R., Nagy L.G., Koehler G., Ransdell A.S., Younus H., Chow J., Chiniquy J., Lipzen A. (2015). Evolution of novel wood decay mechanisms in Agaricales revealed by the genome sequences of *Fistulina hepatica* and *Cylindrobasidium torrendii*. Fungal Genet. Biol..

[31] Bulfer S.L., Scott E.M., Couture J.F., Pillus L., Trievel R.C. (2009). Crystal structure and functional analysis of homocitrate synthase, an essential enzyme in lysine biosynthesis. J. Biol. Chem..

[32] Jogl G., Tong L. (2004). Crystal structure of yeast acetyl-coenzyme A synthetase in complex with AMP. Biochemistry.

[33] Drake E.J., Miller B.R., Shi C., Tarrasch J.T., Sundlov J.A., Allen C.L., Skiniotis G., Aldrich C.C., Gulick A.M. (2016). Structures of two distinct conformations of holo-non-ribosomal peptide synthetases. Nature.

[34] Andi B., Xu H., Cook P.F., West A.H. (2007). Crystal structures of ligand-bound saccharopine dehydrogenase from *Saccharomyces cerevisiae*. Biochemistry.

[35] Burk D.L., Hwang J., Kwok E., Marrone L., Goodfellow V., Dmitrienko G.I., Berghuis A.M. (2007). Structural studies of the final enzyme in the alpha-aminoadipate pathway-saccharopine dehydrogenase from *Saccharomyces cerevisiae*. J. Mol. Biol..

[36] Kumar V.P., Thomas L.M., Bobyk K.D., Andi B., Cook P.F., West A.H. (2012). Evidence in support of lysine 77 and histidine 96 as acid-base catalytic residues in saccharopine dehydrogenase from *Saccharomyces cerevisiae*. Biochemistry.

[37] Krittanawong C., Isath A., Hahn J., Wang Z., Fogg S.E., Bandyopadhyay D., Jneid H., Virani S.S., Tang W.H.W. (2021). Mushroom consumption and cardiovascular health: A systematic review. Am. J. Med..

[38] Storts D.R., Bhattacharjee J.K. (1989). Properties of revertants of Lys2 and Lys5 mutants as well as alpha-aminoadipate-semialdehyde dehydrogenase from *Saccharomyces cerevisiae*. Biochem. Biophys. Res. Commun..

[39] Torruella G., Suga H., Riutort M., Pereto J., Ruiz-Trillo I. (2009). The evolutionary history of lysine biosynthesis pathways within eukaryotes. J. Mol. Evol..

[40] Garnica S., Riess K., Schön M.E., Riess K., Schön M.E. (2016). Divergence times and phylogenetic patterns of Sebacinales, a highly diverse and widespread fungal lineage. PLoS ONE.

[41] Irvin S.D., Bhattacharjee J.K. (1998). A unique fungal lysine biosynthesis enzyme shares a common ancestor with tricarboxylic acid cycle and leucine biosynthetic enzymes found in diverse organisms. J. Mol. Evol..

[42] Ehmann D.E., Gehring A.M., Walsh C.T. (1999). Lysine biosynthesis in *Saccharomyces cerevisiae*: Mechanism of alpha-aminoadipate reductase (Lys2) involves posttranslational phosphopantetheinylation by Lys5. Biochemistry.

