# Supplementary material for: Molecular Evolution of Lysine Biosynthesis in Agaricomycetes
Source: J Fungi (Basel). 2021 Dec 31;8(1):37. doi: 10.3390/jof8010037 (PMC8779187; doi:10.3390/jof8010037)
Supplement: Supplementary file 1 [file jof-08-00037-s001.zip › jof-1510459-SI -supple-final.pdf]

Supplementary data

Molecular evolution of lysine biosynthesis in Agaricomycetes

Zili Song [1, 2], Maoqiang He [1], Ruilin Zhao [1],Landa Qi [3], Guocan Chen [3], Wen-Bing Yin [1, 2*] and Wei Li [1, 2*]

[1] State Key Laboratory of Mycology, Institute of Microbiology, Chinese Academy of Sciences; Beijing 100101, P. R. China
[2] Savaid Medical School, University of Chinese Academy of Sciences, Beijing 100049, P. R. China
[3] Henan Academy of Science Institute of Biology, Zhengzhou 450008, P. R. China

*Correspondence: yinwb@im.ac.cn (W.-B.Y.), liw@im.ac.cn (W. L.); Tel.: +86-10-6480-6170

Table S1. The homologous sequences of HCS used to construct the phylogenetic tree in this study.

Order	Family	Strains	Accession number	Pssm-ID	Cd length	Bit score	Conserved domains Interval	E-value
Agaricales[a]	Tricholomataceae	*Tricholoma matsutake*	KAF8222095.1	163685	262	533.83	64-325	0.00E+00
	Cortinariaceae	*Cortinarius glaucopus*	KAF8802540.1	163685	262	532.68	68-329	0.00E+00
	Pluteaceae	*Pluteus cervinus*	TFK72970.1	163685	262	530.37	66-327	0.00E+00
	Strophariaceae	*Pholiota molesta*	KAF8199646.1	163685	262	531.52	70-331	0.00E+00
	Hymenogastraceae	*Gymnopilus junonius*	KAF8907336.1	163685	262	531.14	72-333	0.00E+00
	Pterulaceae	*Pterula gracilis*	TFK98677.1	163685	262	538.46	61-322	0.00E+00
	Psathyrellaceae	*Coprinopsis marcescibilis*	TFK27163.1	163685	262	532.29	68-329	0.00E+00
	Psathyrellaceae	*Coprinopsis cinerea*	XP_001836353.1	163685	262	535.37	82-343	0.00E+00
	Psathyrellaceae	*Coprinellus angulatus*	KAF6757197.1	163685	262	531.52	70-331	0.00E+00
	Psathyrellaceae	*Coprinellus micaceus*	TEB35407.1	163685	262	531.14	68-329	0.00E+00
	Mycenaceae	*Mycena galopus*	KAF8207270.1	163685	262	532.29	69-330	0.00E+00
	Omphalotaceae	*Gymnopus androsaceus*	KAE9408410.1	163685	262	516.11	64-319	0.00E+00
	Physalacriaceae	*Flammulina velutipes*	AGG53996.1	163685	262	538.07	59-320	0.00E+00
	Physalacriaceae	*Cylindrobasidium torrendii*	KIY65962.1	163685	262	533.83	61-322	0.00E+00
	Physalacriaceae	*Armillaria solidipes*	PBK75543.1	163685	262	530.75	67-328	0.00E+00
	Physalacriaceae	*Armillaria gallica*	PBL03758.1	163685	262	531.14	67-328	0.00E+00
	Omphalotaceae	*Lentinula edodes*	GAW01978.1	163685	262	541.15	593-854	0.00E+00
Polyporales[a]	Polyporaceae	*Trametes coccinea*	OSD06237.1	163685	262	533.45	82-343	0.00E+00
	Polyporaceae	*Trametes versicolor*	EIW63959.1	163685	262	529.6	58-319	0.00E+00
	Polyporaceae	*Dichomitus squalens*	XP_007359783.1	163685	262	533.06	77-338	0.00E+00
	Polyporaceae	*Ganoderma boninense*	VWO94054.1	163685	262	536.53	72-333	0.00E+00
	Polyporaceae	*Polyporus arcularius*	TFK88646.1	163685	262	534.99	73-334	0.00E+00
	Polyporaceae	*Polyporus brumalis*	RDX51874.1	163685	262	534.99	73-334	0.00E+00

	Polyporaceae	*Lentinus tigrinus*	RPD82212.1	163685	262	534.6	67-328	0.00E+00
Russulales[a]	Stereaceae	*Stereum hirsutum*	EIM91005.1	163685	262	529.6	56-317	0.00E+00
	Russulaceae	*Lactarius quietus*	KAF8274751.1	163685	262	529.6	62-323	0.00E+00
	Russulaceae	*Russula ochroleuca*	KAF8485787.1	163685	262	527.67	70-331	0.00E+00
	Russulaceae	*Russula emetica*	KAF8501388.1	163685	262	534.6	65-326	0.00E+00
	Hydnodontaceae	*Peniophora* sp.	KZV64376.1	163685	262	532.68	53-314	0.00E+00
Hymenochaetales[a]	Rickenellaceae	*Rickenella mellea*	TDL17906.1	163685	262	526.9	67-328	0.00E+00
	Hymenochaetaceae	*Pyrrhoderma noxium*	PAV16546.1	163685	262	531.52	63-324	0.00E+00
	Hymenochaetaceae	*Fomitiporia mediterranea*	XP_007262891.1	163685	262	528.06	59-320	0.00E+00
	Hymenochaetaceae	*Sanghuangporus baumii*	OCB85311.1	163685	262	526.9	61-322	0.00E+00
	Schizoporaceae	*Schizopora paradoxa*	KLO12763.1	163685	262	530.75	73-334	0.00E+00
Gomphales[a]	Gomphaceae	*Ramaria rubella*	KAF8591094.1	163685	262	529.98	65-325	0.00E+00
Auriculariales[a]	Auriculariaceae	*Auricularia subglabra*	EJD49060.1	163685	262	538.07	61-322	0.00E+00
	Auriculariaceae	*Exidia glandulosa*	KZV97461.1	163685	262	519.97	42-313	0.00E+00
Cantharellales[a]	Hydnaceae	*Clavulina* sp.	KAF8309935.1	163685	262	535.76	54-314	0.00E+00
	Hydnaceae	*Cantharellus anzutake*	KAF8339827.1	163685	262	523.43	46-306	0.00E+00
Trichosporonales[b]	Dipodascaceae	*Trichosporon asahii*	XP_014181037.1	163685	262	535.76	54-316	0.00E+00
	Trichosporonaceae	*Cutaneotrichosporon oleaginosum*	XP_018282260.1	163685	262	538.07	57-319	0.00E+00
Tremellales[b]	Trimorphomycetaceae	*Saitozyma* sp.	GFZ49222.1	163685	262	518.81	75-327	0.00E+00
Cystofilobasidiales[b]	Mrakiaceae	*Phaffia rhodozyma*	CDZ98541.1	163685	262	532.29	74-336	0.00E+00

Note: "a": Agaricomycetes, "b": Tremellomycetes. Function description: Homocitrate synthase (HCS) catalyzes the condensation of acetyl-CoA and α-ketoglutarate to homocitrate, the first step in the lysine biosynthesis pathway. The HCS of all strains belong to the DRE-TIM metallolyase superfamily (Accession: cd07948).

Table S2. The homologous sequences of HCS used to construct the phylogenetic tree in this study.

Order	Family	Strains	Accession number	Pssm-ID	Cd length	Bit score	Conserved domains Interval	E-value
	Physalacriaceae	*Armillaria gallica*	PBK98805.1	274582	1389	2041.16	14-1425	0.00E+00
	Physalacriaceae	*Armillaria solidipes*	PBK70829.1	274582	1389	2060.42	10-1421	0.00E+00
	Nidulariaceae	*Cyathus striatus*	KAF8999093.1	274582	1389	1879.76	8-1348	0.00E+00
	Psathyrellaceae	*Coprinellus angulatus*	KAF6757844.1	274582	1389	2028.45	8-1435	0.00E+00
	Psathyrellaceae	*Coprinellus micaceus*	TEB29768.1	274582	1389	2062.73	331-1750	0.00E+00
	Amanitaceae	*Amanita rubescens*	KAF8345359.1	274582	1389	2040.39	8-1426	0.00E+00
	Tricholomataceae	*Infundibulicybe gibba*	KAF8886556.1	274582	1389	1954.49	8-1359	0.00E+00
	Agaricaceae	*Macrolepiota fuliginosa*	KAF9447151.1	274582	1389	2046.94	10-1428	0.00E+00
	Pluteaceae	*Pluteus cervinus*	TFK71908.1	274582	1389	2032.69	8-1427	0.00E+00
	Nidulariaceae	*Crucibulum laeve*	TFK37590.1	274582	1389	1956.8	8-1393	0.00E+00
	Crassisporiaceae	*Crassisporium funariophilum*	KAF8157006.1	274582	1389	2062.35	8-1427	0.00E+00
Agaricales[a]	Strophariaceae	*Agrocybe pediades*	KAF9564472.1	274582	1389	2056.57	8-1415	0.00E+00
	Hymenogastraceae	*Flammula alnicola*	KAF8970209.1	274582	1389	2034.23	8-1430	0.00E+00
	Strophariaceae	*Pholiota conissans*	KAF9482698.1	274582	1389	2055.03	8-1427	0.00E+00
	Strophariaceae	*Pholiota molesta*	KAF8180379.1	274582	1389	1716.44	8-1208	0.00E+00
	Psathyrellaceae	*Panaeolus papilionaceus*	KAF9041288.1	274582	1389	2060.8	12-1431	0.00E+00
	Hydnangiaceae	*Laccaria bicolor*	XP_001879618.1	274582	1389	2059.65	8-1419	0.00E+00
	Lyophyllaceae	*Hypsizygus marmoreus*	RDB30310.1	274582	1389	2051.56	8-1420	0.00E+00
	Lyophyllaceae	*Lyophyllum atratum*	KAF8067844.1	274582	1389	2050.79	8-1414	0.00E+00
	Tricholomataceae	*Tricholoma matsutake*	KAF8223431.1	274582	1389	2025.37	12-1407	0.00E+00
	Tricholomataceae	*Lepista nuda*	KAF9459773.1	274582	1389	2036.54	8-1416	0.00E+00
	Mycenaceae	*Mycena indigotica*	XP_037213600.1	274582	1389	2023.44	429-1847	0.00E+00
	Mycenaceae	*Mycena chlorophos*	KAF7300613.1	274582	1389	2013.81	10-1400	0.00E+00
Boletales[a]	Paxillaceae	*Gyrodon lividus*	KAF9226319.1	274582	1389	1984.15	8-1437	0.00E+00

	Boletaceae	*Xerocomus badius*	KAF8553785.1	274582	1389	2001.1	8-1437	0.00E+00
	Suillaceae	*Suillus brevipes*	KAF3875411.1	274582	1389	1989.54	8-1432	0.00E+00
	Gloeophyllaceae	*Neolentinus lepideus*	KZT28090.1	274582	1389	2055.41	12-1418	0.00E+00
Gloeophyllales[a]	Gloeophyllaceae	*Heliocybe sulcata*	TFK49407.1	274582	1389	2090.85	11-1421	0.00E+00
	Gloeophyllaceae	*Gloeophyllum trabeum*	XP_007867945.1	274582	1389	2076.21	12-1424	0.00E+00
	Laetiporaceae	*Laetiporus sulphureus*	KZT10254.1	274582	1389	2066.97	8-1426	0.00E+00
	Fomitopsidaceae	*Daedalea quercina*	KZT70440.1	274582	1389	2046.17	8-1420	0.00E+00
Polyporales[a]	Polyporaceae	*Lentinus tigrinus*	RPD65725.1	274582	1389	2092.78	11-1428	0.00E+00
	Polyporaceae	*Polyporus brumalis*	RDX54732.1	274582	1389	2112.04	10-1432	0.00E+00
	Polyporaceae	*Dichomitus squalens*	TBU61097.1	274582	1389	2053.1	11-1427	0.00E+00
	Stereaceae	*Stereum hirsutum*	XP_007309010.1	274582	1389	2062.35	11-1425	0.00E+00
	Hericiaceae	*Dentipellis* sp.	KAA1473026.1	274582	1389	2066.97	11-1418	0.00E+00
	Russulaceae	*Russula ochroleuca*	KAF8480159.1	274582	1389	1982.22	11-1423	0.00E+00
Russulales[a]	Russulaceae	*Russula emetica*	KAF8491911.1	274582	1389	1949.87	11-1414	0.00E+00
	Russulaceae	*Lactarius quietus*	KAF8271296.1	274582	1389	1972.21	11-1419	0.00E+00
	Peniophoraceae	*Peniophora* sp.	KZV74202.1	274582	1389	2024.6	11-1410	0.00E+00
Cantharellales[a]	Hydnaceae	*Clavulina* sp.	KAF8318757.1	274582	1389	2026.52	11-1409	0.00E+00
Cystofilobasidiales[b]	Mrakiaceae	*Phaffia rhodozyma*	CDZ96380.1	274582	1389	2067.35	13-1437	0.00E+00

Note: "a": Agaricomycetes, "b": Tremellomycetes. Function description: α-Aminoadipate reductase (AAR) catalyzes the reduction of α-aminoadipate to semialdehyde. The AAR of all strains belong to the alpha_am_amid superfamily (Accession: cl27680).

Table S3. The homologous sequences of HCS used to construct the phylogenetic tree in this study.

Order	Family	Strains	GenBank No.	Pssm-ID	Cd Length	Bit Score	Accession	Interval	E-value
Agaricales [a]	Lyophyllaceae	*Hypsizygus marmoreus*	RDB29103.1	240664	351	535.66	cd12188	33-385	0.00E+00
	Tricholomataceae	*Tricholoma matsutake*	KAF8223463.1	240664	351	533.73	cd12188	6-360	0.00E+00
	Tricholomataceae	*Lepista nuda*	KAF9457093.1	240664	351	513.7	cd12188	5-355	0.00E+00
	Mycenaceae	*Mycena galopus*	KAF8213335.1	240664	351	550.3	cd12188	4-360	0.00E+00
	Marasmiaceae	*Marasmius fiardii*	KAF9270414.1	240664	351	535.27	cd12188	4-360	0.00E+00
	Pleurotaceae	*Pleurotus ostreatus*	XP_036634909.1	240664	351	555.69	cd12188	4-360	0.00E+00
	Pleurotaceae	*Pleurotus pulmonarius*	KAF4605402.1	240664	351	550.68	cd12188	4-360	0.00E+00
	Agaricaceae	*Macrolepiota fuliginosa*	KAF9454889.1	240664	351	557.23	cd12188	4-361	0.00E+00
	Agaricaceae	*Leucoagaricus* sp.	KXN89843.1	240664	351	563.01	cd12188	4-365	0.00E+00
	Psathyrellaceae	*Coprinopsis marcescibilis*	TFK30628.1	240664	351	541.05	cd12188	4-360	0.00E+00
	Marasmiaceae	*Moniliophthora roreri*	ESK98395.1	240664	351	545.29	cd12188	4-359	0.00E+00
	Crassisporiaceae	*Crassisporium funariophilum*	KAF8167912.1	240664	351	536.82	cd12188	4-360	0.00E+00
	Nidulariaceae	*Crucibulum laeve*	TFK40972.1	240664	351	554.15	cd12188	4-360	0.00E+00
	Bolbitiaceae	*Panaeolus papilionaceus*	KAF9056365.1	240664	351	556.46	cd12188	4-360	0.00E+00
	Cortinariaceae	*Gymnopilus junonius*	KAF8912853.1	240664	351	538.36	cd12188	39-395	0.00E+00
	Physalacriaceae	*Flammulina velutipes*	QCX08356.1	240664	351	569.17	cd12188	4-360	0.00E+00
	Physalacriaceae	*Armillaria gallica*	PBL00798.1	240664	351	573.41	cd12188	4-360	0.00E+00
	Physalacriaceae	*Armillaria solidipes*	PBK77012.1	240664	351	573.41	cd12188	4-360	0.00E+00
	Physalacriaceae	*Guyanagaster necrorhizus*	KAG7450318.1	240664	351	571.1	cd12188	4-360	0.00E+00
Boletales [a]	Suillaceae	*Suillus spraguei*	KAG2368229.1	240664	351	555.3	cd12188	5-358	0.00E+00
	Suillaceae	*Suillus decipiens*	KAG2077096.1	240664	351	554.53	cd12188	6-358	0.00E+00
	Suillaceae	*Suillus clintonianus*	XP_041206159.1	240664	351	556.08	cd12188	6-358	0.00E+00
	Suillaceae	*Suillus ampliporus*	KAG0709064.1	240664	351	559.16	cd12188	6-358	0.00E+00

	Rhizopogonaceae	*Rhizopogon vinicolor*	OAX44812.1	240664	351	526.03	cd12188	6-358	0.00E+00
	Suillaceae	*Suillus paluster*	XP_041183794.1	240664	351	551.45	cd12188	6-353	0.00E+00
	Suillaceae	*Suillus brevipes*	KAG2754539.1	240664	351	561.47	cd12188	6-358	0.00E+00
	Suillaceae	*Suillus occidentalis*	KAG1773126.1	240664	351	552	cd12188	6-358	0.00E+00
	Suillaceae	*Suillus placidus*	KAG1783737.1	240664	351	551.07	cd12188	6-361	0.00E+00
	Suillaceae	*Suillus americanus*	KAG2044207.1	240664	351	554.92	cd12188	6-358	0.00E+00
	Suillaceae	*Suillus subalutaceus*	XP_041248104.1	240664	351	556.85	cd12188	6-358	0.00E+00
	Suillaceae	*Suillus subaureus*	XP_041199417.1	240664	351	557.23	cd12188	6-358	0.00E+00
	Suillaceae	*Suillus cothurnatus*	KAG2149799.1	240664	351	560.7	cd12188	6-358	0.00E+00
	Suillaceae	*Suillus lakei*	KAG1757371.1	240664	351	530.27	cd12188	6-351	0.00E+00
	Suillaceae	*Suillus bovinus*	XP_041307276.1	240664	351	559.54	cd12188	5-358	0.00E+00
	Suillaceae	*Suillus variegatus*	KAG1834565.1	240664	351	562.62	cd12188	5-358	0.00E+00
	Suillaceae	*Suillus tomentosus*	KAG1866257.1	240664	351	557.23	cd12188	6-358	0.00E+00
	Suillaceae	*Suillus hirtellus*	KAG2062054.1	240664	351	561.85	cd12188	5-358	0.00E+00
	Suillaceae	*Suillus discolor*	XP_041298324.1	240664	351	561.08	cd12188	5-358	0.00E+00
	Suillaceae	*Suillus plorans*	XP_041164869.1	240664	351	559.16	cd12188	6-358	0.00E+00
	Suillaceae	*Suillus fuscotomentosus*	XP_041220431.1	240664	351	564.16	cd12188	5-358	0.00E+00
	Boletaceae	*Xerocomus badius*	KAF8551619.1	240664	351	548.37	cd12188	4-359	0.00E+00
	Boletaceae	*Boletus reticuloceps*	KAG6376707.1	240664	351	539.9	cd12188	4-359	0.00E+00
	Gyrodontaceae	*Gyrodon lividus*	KAF9229508.1	240664	351	524.1	cd12188	7-362	0.00E+00
	Paxillaceae	*Paxillus involutus*	KIJ21575.1	240664	351	537.97	cd12188	4-359	0.00E+00
	Paxillaceae	*Paxillus ammoniavirescens*	KAF8845261.1	240664	351	537.2	cd12188	4-359	0.00E+00
	Polyporaceae	*Polyporus brumalis*	RDX56030.1	240664	351	582.27	cd12188	6-372	0.00E+00
Polyporales [a]	Polyporaceae	*Polyporus arcularius*	TFK94363.1	240664	351	578.8	cd12188	6-372	0.00E+00
	Polyporaceae	*Lentinus tigrinus*	RPD63103.1	240664	351	577.26	cd12188	6-360	0.00E+00

	Polyporaceae	*Dichomitus squalens*	TBU64909.1	240664	351	571.87	cd12188	6-360	0.00E+00
	Fomitopsidaceae	*Daedalea quercina*	KZT74771.1	240664	351	558.77	cd12188	6-360	0.00E+00
	Polyporaceae	*Trametes pubescens*	OJT12634.1	240664	351	572.64	cd12188	6-367	0.00E+00
	Polyporaceae	*Trametes versicolor*	XP_008032403.1	240664	351	570.71	cd12188	6-367	0.00E+00
	Polyporaceae	*Trametes coccinea*	OSD06069.1	240664	351	592.67	cd12188	6-360	0.00E+00
Tremellales[b]	Cryptococcaceae	*Cryptococcus wingfieldii*	XP_019027901.1	240664	351	514.47	cd12188	21-380	0.00E+00
	Cryptococcaceae	*Cryptococcus neoformans*	OWZ45351.1	240664	351	527.96	cd12188	21-381	0.00E+00
	Cryptococcaceae	*Cryptococcus gattii*	KIR47068.1	240664	351	529.5	cd12188	21-381	0.00E+00
	Trimorphomycetaceae	*Saitozyma podzolica*	RSH89270.1	240664	351	513.7	cd12188	21-382	0.00E+00
	Trimorphomycetaceae	*Saitozyma* sp.	GFZ45358.1	240664	351	521.02	cd12188	21-382	0.00E+00
	Naemateliaceae	*Naematelia encephala*	ORY31427.1	240664	351	518.71	cd12188	30-391	0.00E+00
	Cryptococcaceae	*Kwoniella mangroviensis*	XP_019000803.1	240664	351	520.25	cd12188	21-381	0.00E+00
	Cryptococcaceae	*Kwoniella bestiolae*	XP_019045793.1	240664	351	519.48	cd12188	21-381	0.00E+00
	Cryptococcaceae	*Kwoniella dejecticola*	XP_018262506.1	240664	351	506	cd12188	21-381	0.00E+00
	Cryptococcaceae	*Kwoniella heveanensis*	OCF30597.1	240664	351	516.4	cd12188	21-381	0.00E+00
Trichosporonales[b]	Trichosporonaceae	*Apiotrichum porosum*	XP_028474516.1	240664	351	532.58	cd12188	7-369	0.00E+00
	Trichosporonaceae	*Trichosporon asahii*	XP_014180072.1	240664	351	532.58	cd12188	8-368	0.00E+00
Cystofilobasidiales[b]	Mrakiaceae	*Phaffia rhodozyma*	CDZ96774.1	240664	351	516.4	cd12188	7-364	0.00E+00

Note: "a": Agaricomycetes, "b": Tremellomycetes. Function description: Saccharopine dehydrogenase (SDH) catalyzes the cleavage of saccharopine to α-ketoglutarate and L-lysine, the final step in the lysine biosynthesis pathway. The SDH of all strains belong to the PLN02819 superfamily (Accession: cl33572).

Table S4. The sequences used to construct the phylogenetic tree of species in this study.

Order	Family	Species	Strains	LSU	SSU	RPB1	RPB2	ITS(5.8S)	EF1-α
Agaricales[a]	Amanitaceae	*Amanita rubescens*	HKAS92040	MH486816.1	—	—	MH486227.1	MH508559.1	MH509043.1
	Amanitaceae	*Macrolepiota fuliginosa*	TO AFM11	—	—	—	—	HM246502.1	—
	Amanitaceae	*Leucoagaricus* sp.	177275	MT669247.1	—	—	—	MT669115.1	—
	Amanitaceae	*Panaeolus papilionaceus*	DNA1940	KF830082.1	KF830073.1	—	KF830065.1	KF830093.1	—
	Crassisporiaceae	*Crassisporium funariophilum*	366861	—	—	—	—	MT703796.1	—
	Cortinariaceae	*Cortinarius glaucopus*	TUB 011414	—	—	EU088237.1	—	AY174787.1	—
	Hymenogastraceae	*Flammula alnicola*	AFTOL-ID 1501	DQ457666.1	DQ113916.1	DQ447900.1	DQ472714.1	—	—
		Gymnopilus junonius	CBS 420.50	MH868214.1	—	—	—	MH856698.1	—
	Nidulariaceae	*Crucibulum laeve*	TUB 011564	DQ071771.1	—	DQ067950.1	—	DQ071701.2	—
	Lyophyllaceae	*Hypsizygus marmoreus*	3144	—	—	—	—	FJ609282.1	—
	Lyophyllaceae	*Lyophyllum atratum*	CBS709.87	—	—	EF421033.1	EF420999.1	—	EF421065.1
	Marasmiaceae	*Marasmius fiardii*	—	—	—	—	—	—	—
	Marasmiaceae	*Moniliophthora roreri*	C21	AY916744.1	AY916745.1	AY916747.1	—	AY916746.1	AY916748.1
	Mycenaceae	*Mycena indigotica*	WEI 16-475	MF993024.1	—	—	—	MF993026.1	—
	Mycenaceae	*Mycena chlorophos*	ACL271	—	—	—	KJ406371.1	KJ206986.1	—
	Mycenaceae	*Mycena galopus*	CBS 182.46	MH867683.1	—	—	—	MH856158.1	—
	Nidulariaceae	*Cyathus striatus*	TUB 011565	DQ071742.2	—	DQ068021.1	—	—	—
	Omphalotaceae	*Gymnopus androsaceus*	CBS 241.53	MH868715.1	—	—	—	MH857176.1	—
	Omphalotaceae	*Lentinula edodes*	TMI1941	AF261557	AF082686	—	—	AY636053	—
	Physalacriaceae	*Armillaria gallica*	HKAS85517	—	—	—	—	KT822312.1	KT822409.1
	Physalacriaceae	*Armillaria solidipes*	W1-9	JF895919.1	—	—	JF895886.1	—	JF895853.1

	Physalacriaceae	*Cylindrobasidium torrendii*	CBS 125844	MH875265.1	—	—	—	MH863803.1	—
	Physalacriaceae	*Flammulina velutipes*	AFTOL_ID_558	AY639883	AY665781	AY858966	AY786055	AY854073	—
	Physalacriaceae	*Guyanagaster necrorhizus*	G31.4	KU254227.1	—	—	—	KU170950.1	KU289110.1
	Psathyrellaceae	*Coprinellus angulatus*	CBS 175.51	MH868315.1	—	—	—	MH856799.1	—
	Psathyrellaceae	*Coprinellus micaceus*	SZMC-NL-4253	—	JN939889.1	—	—	JN943116.1	—
	Psathyrellaceae	*Coprinopsis cinerea*	CBS 126970	MH875802.1	—	—	—	MH864360.1	—
	Psathyrellaceae	*Coprinopsis marcescibilis*	CBS 302.47	MH867797.1	—	—	—	MH856262.1	—
	Pluteaceae	*Pluteus cervinus*	FO 46619	DQ071729.2	—	DQ067955.1	—	—	—
	Pleurotaceae	*Pleurotus ostreatus*	6689	—	—	—	MK810572.1	AY450345.1	MK810672.1
	Pleurotaceae	*Pleurotus pulmonarius*	ZRL20151671	KY418877.1	KY418943.1	—	KY419022.1	LT716061.1	KY419073.1
	Pterulaceae	*Pterula gracilis*	IO.14.142 (S)	MT232310.1	MT232498.1	—	—	MT232356.1	MT242358.1
	Strophariaceae	*Agrocybe pediades*	AFTOL-ID 1493	DQ110872.1	DQ113915.1	—	—	DQ484057.1	—
	Strophariaceae	*Pholiota conissans*	CBS 175.47	MH867735.1	—	—	—	MH856205.1	—
	Strophariaceae	*Pholiota molesta*	MTS4953a	—	—	—	MG923696.1	MG735309.1	MN311971.1
	Tricholomataceae	*Infundibulicybe gibba*	AFTOL-ID 1508	DQ457682.1	DQ115780.1	DQ447913.1	DQ472727.2	DQ490635.1	—
	Tricholomataceae	*Laccaria bicolor*	KA13-0253	MG519570.1	—	—	MG551599.1	MG519524.1	MG551636.1
	Tricholomataceae	*Lepista nuda*	11CA041	KJ021705.1	KJ021692.1	—	KJ136110.1	—	MG702630.1
	Tricholomataceae	*Tricholoma matsutake*	Tm029	—	—	—	—	AB559004.1	AB699735.1
Boletales[a]	Paxillaceae	*Gyrodon lividus*	REG Gl1	—	DQ534681.1	—	GU187786.1	DQ534568.1	GU187701.1
	Paxillaceae	*Paxillus involutus*	Bel10.4	—	—	—	JQ436854.1	KF261366.1	KF261504.1
	Paxillaceae	*Paxillus ammoniavirescens*	Cas12.1	—	—	—	—	KF261380.1	KF261518.1
	Boletaceae	*Xerocomus badius*	MB 03-098a	KF030355	—	KF030393	—	—	KF030423

	Boletus reticuloceps	HKAS62910	JN563843.1	—	JN563862.1	—	JN563884.1	—
Suillaceae	*Rhizopogon vinicolor*	JMT20787	—	—	—	—	AF062941.1	—
Suillaceae	*Suillus brevipes*	F1187371	KU721323.1	—	KU852270.1	KU852302.1	KU721224.1	KU721760.1
Suillaceae	*Suillus spraguei*	TDB-638	KU721505.1	—	KU852201.1	KU852358.1	KU721527.1	KU721721.1
Suillaceae	*Suillus decipiens*	BKr1029002	KU721521.1	—	—	—	KU721495.1	KU721709.1
Suillaceae	*Suillus clintonianus*	07.08.30-19	—	—	—	—	KU059568.1	—
Suillaceae	*Suillus ampliporus*	ACD0177	—	—	—	—	MZ919172.1	—
Suillaceae	*Suillus paluster*	HKAS:63134	KU721407.1	—	KU852251.1	KU852371.1	KU721255.1	KU721586.1
Suillaceae	*Suillus occidentalis*	Mushroom Observer 283944	—	—	—	—	MT437070.1	—
Suillaceae	*Suillus placidus*	TENN:062310	KU721389.1	—	KU852189.1	KU852364.1	KU721274.1	KU721558.1
Suillaceae	*Suillus americanus*	C0075059F	KY489971.1	—	KU852261.1	KU852351.1	KU663184.1	KU663205.1
Suillaceae	*Suillus subalutaceus*	MN175	—	—	—	—	KX230630.1	—
Suillaceae	*Suillus subaureus*	F1189253	KU721365.1	—	KU852203.1	KU852356.1	KU721171.1	KU721699.1
Suillaceae	*Suillus cothurnatus*	MA-Fungi 47683	—	—	—	—	AJ419218.1	—
Suillaceae	*Suillus lakei*	RZ120208	KU721361.1	—	KU852242.1	KU852374.1	KU721189.1	KU721589.1
Suillaceae	*Suillus bovinus*	KM164971	KU721292.1	—	KU852269.1	KU852336.1	KU721203.1	KU721733.1
Suillaceae	*Suillus variegatus*	DG86	—	—	—	—	JQ888210.1	—
Suillaceae	*Suillus tomentosus*	F1186917	KU721301.1	—	KU852205.1	KU852345.1	KU721157.1	KU721748.1
Suillaceae	*Suillus hirtellus*	ADW:0118	KX171000.1	—	—	KX171007.1	KU721152.1	KX171004.1
Suillaceae	*Suillus discolor*	MICH 341813	—	—	—	—	KX213790.1	—
Suillaceae	*Suillus plorans*	HKAS:63225	KU721313.1	—	—	—	KU721165.1	KU721738.1
Suillaceae	*Suillus fuscotomentosus*	BW6	KU721309.1	—	KU852283.1	KU852340.1	KU721158.1	KU721747.1
Gloeophyllaceae	*Neolentinus lepideus*	DAOM:208668	HM536077.1	HM536078.1	—	HM536121.1	—	HM536122.1

Gloeophyllales[a]	Gloeophyllaceae	*Heliocybe sulcata*	IBUG 9930	HM536069	HM536070	—	HM536114	HM536095	HM536115
	Gloeophyllaceae	*Gloeophyllum trabeum*	1320	HM536067.1	HM536068.1	—	HM536112.1	HM536094.1	HM536113.1
Polyporales[a]	Laetiporaceae	*Laetiporus sulphureus*	Dai 12154	KF951302	KR605924	—	KR610841	KF951295	KR610752
	Fomitopsidaceae	*Daedalea quercina*	Dai 12659	KP171230.1	KR605887.1	—	KR610810.1	KP171208.1	KR610719.1
	Polyporaceae	*Lentinus tigrinus*	DSH92D787	—	—	KP325689.1	—	KP283488.1	—
	Polyporaceae	*Polyporus arcularius*	Cui 6450	KX900098.1	KX905089.1	KX900376.1	KX900299.1	KX899967.1	KX900326.1
	Polyporaceae	*Polyporus brumalis*	Cui 2769	KX851650.1	KX851726.1	KX851748.1	KX851759.1	KX851595.1	KX851775.1
	Polyporaceae	*Trametes coccinea*	Cui 7096	—	KX880718.1	KX880822.1	KX880857.1	—	KX880892.1
	Polyporaceae	*Trametes versicolor*	ZRL20151477	KY418903	KY418958	KY418984	KY419041	LT716079	KY419091
	Polyporaceae	*Trametes pubescens*	HHB13585sp	—	—	KF573164.1	KF573117.1	JN164947.1	KF573060.1
	Polyporaceae	*Ganoderma boninense*	WD 2085	—	—	KJ143945.1	KJ143965.1	KJ143906.1	KJ143925.1
	Polyporaceae	*Dichomitus squalens*	Cui 9639	—	KX838404.1	KX838471.1	KX838478.1	—	KX838436.1
	Stereaceae	*Stereum hirsutum*	AFTOL-ID 492	—	—	AY864885.1	AY218520.2	AY854063.1	AY885159.1
Russulales[a]	Hericiaceae	*Dentipellis* sp.	Dai 18768	MH571700.1	—	—	—	MH571703.1	—
	Russulaceae	*Russula ochroleuca*	FH12211	KT933857.1	—	KT957368.1	KT933928.1	KT933996.1	—
	Russulaceae	*Russula emetica*	635/JMT39.08092228	KU237578.1	KU237426.1	KU237721.1	KU237864.1	—	—
	Russulaceae	*Lactarius quietus*	KW138	—	—	—	KR025739.1	KT165231.1	—
	Peniophoraceae	*Peniophora* sp.	HUT104	—	—	—	—	KU377265.1	—
Hymenochaetales[a]	Rickenellaceae	*Rickenella mellea*	CBS 581.87	MH873796.1	—	—	—	MH862107.1	—
	Hymenochaetaceae	*Pyrrhoderma noxium*	UO 7	MG734465.1	—	—	MH398393.1	MG645087.1	MH398488.1
	Hymenochaetaceae	*Fomitiporia mediterranea*	AFTOL-ID 688	AY684157.1	AY662664.1	AY864870.1	AY803748.1	AY854080.1	AY885149.1
	Hymenochaetaceae	*Sanghuangporus baumii*	Dai 17133	MH101002.1	—	MH101029.1	MH101237.1	—	MH101118.1

	Schizoporaceae	*Schizopora paradoxa*	CBS 319.53	MH868758.1	—	—	—	MH857220.1	—
Gomphales[a]	Gomphaceae	*Ramaria rubella*	AFTOL-ID 724	AY645057.1	AY707095.1	AY864866.1	AY786064.1	AY854078.1	AY883435.1
Auriculariales[a]	Auriculariaceae	*Auricularia subglabra*	Dai 17403	MH213419.1	—	—	MH213448.1	MH213382.1	—
		Exidia glandulosa	Dai 18024	MH213426.1	—	—	MH213457.1	MH213394.1	—
Cantharellales[a]	Hydnaceae	*Clavulina* sp.	MB03-034	AY745694.1	AY757265.1	—	DQ366286.1	DQ202266.1	DQ028589.1
Tremellales[b]	Trimorphomycetaceae	*Saitozyma podzolica*	CBS_6819	—	—	—	—	AF444321.1	—
	Trimorphomycetaceae	*Saitozyma pseudoflava*	XZ200A1	—	—	—	—	MK0502884.1	—
	Trimorphomycetaceae	*Saitozyma* sp.	XZ200A1	—	—	MK849251.1	MK849387.1	—	MK849114.1
	Naemateliaceae	*Naematelia encephala*	CBS6968	—	—	—	—	AF410474.1	—
	Naemateliaceae	*Tremella encephala*	CBS6968	—	KF036698.1	KF036525.1	KF036941.1	—	KF037203.1
	Cryptococcaceae	*Kwoniella dejecticola*	CBS:10117	KY108197.1	—	—	—	KY103929.1	—
	Cryptococcaceae	*Kwoniella bestiolae*	CBS 10118	KY108196.1	—	—	—	KY103928.1	—
	Cryptococcaceae	*Kwoniella mangrovensis*	CBS 8507	FJ534912.1	FJ534897.1	FJ534928.1	FJ534943.1	FJ534882.1	FJ534866.1
	Cryptococcaceae	*Kwoniella heveanensis*	CBS 569	FJ534905.1	FJ534891.1	FJ534921.1	—	FJ534875.1	FJ534860.1
	Cryptococcaceae	*Cryptococcus gattii*	CBS:10514	KY107017.1	—	—	—	KY102661.1	—
	Cryptococcaceae	*Filobasidiella neoformans*	CBS 132	AF075484.1	—	—	—	—	—
	Cryptococcaceae	*Cryptococcus neoformans*	CBS 132	—	HQ596559	KF036472.1	KF036886	AF444326.1	KF037151.1
	Cryptococcaceae	*Tsuchiyaea wingfieldii*	CBS7118	—	—	KF036577.1	KF036994.1	EF211246.1	KF037250.1

Trichosporonales[b]	Trichosporonaceae	*Cutaneotrichosporon oleaginosum*	R97187	—	—	—	—	MK268120.1	—
	Trichosporonaceae	*Apiotrichum porosum*	AMF086	MN307946.1	—	—	—	MN309722.1	—
	Trichosporonaceae	*Trichosporon asahii*	CBS 2479	—	—	KF036539.1	KF036955.1	—	KF037216.1
	Trichosporonaceae	*Cryptococcus podzolicus*	CBS6819	AF075481.1	AB032645.1	KF036396.1	KF036810.1	—	KF037081.1
Cystofilobasidiales[b]	Mrakiaceae	*Phaffia rhodozyma*	CBS5905	—	KF036689.1	—	KF036933.1	DQ904246.1	KF037195.1

Note: "a": Agaricomycetes, "b": Tremellomycetes. "-": Not sequence found in NCBI. LSU: Large subunit, SSU: Small subunit, RPB1: RNA Polymerase I, RPB2: RNA Polymerase II, ITS (5.8S): Internal transcribed spacer in 5.8S rRNA, EF1-α: Translation elongation factor 1-alpha.

Table S5. Characteristics of the predicted secondary structures of HCS, AAR and SDH in Agaricomycetes.

Strains	Alpha helix (%)			Extended strand (%)			Beta turn (%)			Random coil (%)		
	HCS	AAR	SDH	HCS	AAR	SDH	HCS	AAR	SDH	HCS	AAR	SDH
Armillaria gallica	44.49(210)	36.56(521)	31.42(115)	12.50(59)	15.23(217)	19.67(72)	6.14(29)	4.70 (67)	4.64(17)	36.86(174)	43.51(620)	44.26(162)
Armillaria solidipes	42.58(201)	37.51(533)	31.97(117)	12.92(61)	14.78(210)	19.95(73)	5.7 (27)	4.50(64)	4.10(15)	38.77(183)	43.21(614)	43.99(161)
Guyanagaster necrorhizus	—	—	32.07(118)	—	—	19.84(73)	—	—	4.08(15)	—	—	44.02(162)
Cyathus striatus	—	37.73(509)	—	—	14.83(200)	—	—	5.19(70)	—	—	42.25(570)	—
Coprinellus angulatus	43.72(209)	35.51(435)	—	11.72(56)	15.76(193)	—	5.23(25)	5.88 (72)	—	39.33(188)	42.86(525)	—
Coprinellus micaceus	43.70(208)	34.67(607)	—	10.92(52)	16.16(283)	—	5.67(27)	5.43(95)	—	39.71(189)	43.75(766)	—
Coprinopsis cinerea	41.63(204)	—	—	11.63(57)	—	—	5.10(25)	—	—	41.63(204)	—	—
Coprinopsis marcescibilis	44.12(210)	—	32.52(120)	12.39(59)	—	19.78(73)	5.46(26)	—	5.15(19)	38.03(181)	—	42.55(157)
Cortinarius glaucopus	43.71(205)	—	—	12.37(58)	—	—	5.12(24)	—	—	38.81(182)	—	—
Amanita rubescens	—	37.45(534)	—	—	14.94(213)	—	—	4.70 (67)	—	—	42.92(612)	—
Infundibulicybe gibba	—	37.15(516)	—	—	14.61(203)	—	—	5.04 (70)	—	—	43.20(600)	—
Macrolepiota fuliginosa	—	37.02(529)	31.98(118)	—	14.63(209)	19.51(72)	—	5.04 (72)	4.61(17)	—	43.32(619)	43.9 (162)
Pluteus cervinus	43.31(204)	37.25(532)	—	12.95(61)	14.92(213)	—	5.73(27)	4.90 (70)	—	38.00(179)	42.93(613)	—
Crucibulum laeve	—	36.37(507)	32.88(121)	—	14.85(207)	18.21(67)	—	4.81 (67)	4.62(17)	—	43.97(613)	44.29(163)
Crassisporium funariophilum	—	36.97(528)	32.34(119)	—	14.85(212)	19.02(70)	—	4.90 (70)	4.35(16)	—	43.28(618)	44.29(163)
Agrocybe pediades	—	37.01(524)	—	—	15.25(216)	—	—	5.08 (72)	—	—	42.66(604)	—
Flammula alnicola	—	37.53(537)	—	—	14.68(210)	—	—	5.03 (72)	—	—	42.77(612)	—
Flammulina velutipes	43.75(203)	—	30.98(114)	13.15(61)	—	19.02(70)	6.90(32)	—	3.53(13)	36.21(168)	—	46.47(171)
Cylindrobasidium torrendii	41.47(192)	—	—	12.96(60)	—	—	6.05(28)	—	—	39.52(183)	—	—
Pterula gracilis	43.23(198)	—	—	12.88(59)	—	—	5.90(27)	—	—	37.99(174)	—	—
Gymnopilus junonius	41.04(197)	—	28.78(116)	13.75(66)	—	18.36(74)	5.83(28)	—	4.22(17)	39.38(189)	—	48.64(196)
Gymnopus androsaceus	42.92(200)	—	—	12.23(57)	—	—	5.79(27)	—	—	39.06(182)	—	—
Pholiota conissans	—	36.83(526)	—	—	14.50(207)	—	—	4.69 (67)	—	—	43.98(628)	—

Pholiota molesta	42.47(203)	36.49(451)	—	11.51(55)	15.86(196)	—	5.65(27)	4.45 (55)	—	40.38(193)	43.20(534)	—
Panaeolus papilionaceus	—	37.64(539)	31.79(117)	—	14.66(210)	19.02(70)	—	4.75 (68)	3.53(13)	—	42.95(615)	45.65(168)
Laccaria bicolor	—	38.66(549)	—	—	14.44(205)	—	—	4.30 (61)	—	—	42.61(605)	—
Lentinula edodes	45.85(210)	—	—	13.10(60)	—	—	6.33(29)	—	—	34.72(159)	—	—
Hypsizygus marmoreus	—	36.73(522)	—	—	14.92(212)	19.85(78)	—	4.79 (68)	3.82(15)	—	43.56(619)	46.31(182)
Lyophyllum atratum	—	37.84(535)	—	—	15.21(215)	—	—	4.88 (69)	—	—	42.08(595)	—
Tricholoma matsutake	44.89(211)	36.66(521)	32.34(119)	12.98(61)	14.85(211)	19.29(71)	6.60(31)	4.86 (69)	5.16(19)	35.53(167)	43.63(620)	43.21(159)
Lepista nuda	—	36.51(517)	32.88(121)	—	15.11(214)	19.29(71)	—	4.80 (68)	3.80(14)	—	43.57(617)	44.02(162)
Pleurotus pulmonarius	—	—	30.27(112)	—	—	18.92(70)	—	—	3.51(13)	—	—	47.3 (175)
Pleurotus ostreatus	—	—	33.78(125)	—	—	20.27(75)	—	—	3.51(13)	—	—	42.43(157)
Mycena indigotica	—	35.08(648)	—	—	16.24(300)	—	—	5.63(104)	—	—	43.04(795)	—
Mycena chlorophos	—	38.14(534)	—	—	15.00(210)	—	—	4.79 (67)	—	—	42.07(589)	—
Mycena galopus	44.28(209)	—	31.79(117)	12.08(57)	—	19.29(71)	5.51(26)	—	4.89(18)	38.14(180)	—	44.02(162)
Leucoagaricus sp.	—	—	30.83(115)	—	—	18.77(70)	—	—	4.83(18)	—	—	45.58(170)
Moniliophthora roreri	—	—	32.61(120)	—	—	19.84(73)	—	—	3.53(13)	—	—	44.02(162)
Marasmius fiardii	—	—	30.93(116)	—	—	18.67(70)	—	—	4.80(18)	—	—	45.6 (171)
Suillus cothurnatus	—	—	32.25(119)	—	—	19.51(72)	—	—	3.52(13)	—	—	44.72(165)
Suillus clintonianus	—	—	31.79(117)	—	—	19.57(72)	—	—	4.35(16)	—	—	44.29(163)
Suillus subalutaceus	—	—	31.79(117)	—	—	19.02(70)	—	—	4.35(16)	—	—	44.84(165)
Suillus ampliporus	—	—	31.25(115)	—	—	19.29(71)	—	—	4.35(16)	—	—	45.11(166)
Suillus fuscotomentosus	—	—	31.52(116)	—	—	19.84(73)	—	—	4.89(18)	—	—	43.75(161)
Suillus plorans	—	—	32.07(118)	—	—	19.84(73)	—	—	4.35(16)	—	—	43.7 (161)
Suillus americanus	—	—	31.25(115)	—	—	19.84(73)	—	—	3.80(14)	—	—	45.11(166)
Suillus spraguei	—	—	31.52(116)	—	—	19.84(73)	—	—	4.62(17)	—	—	44.02(162)
Suillus decipiens	—	—	32.34(119)	—	—	19.29(71)	—	—	4.35(16)	—	—	44.02(162)
Suillus bovinus	—	—	32.61(120)	—	—	18.75(69)	—	—	4.08(15)	—	—	44.57(164)

Suillus tomentosus	—	—	32.88(121)	—	—	19.29(71)	—	—	5.16(19)	—	—	42.66(157)
Suillus hirtellus	—	—	31.79(117)	—	—	19.84(73)	—	—	4.08(15)	—	—	44.29(163)
Suillus discolor	—	—	32.07(118)	—	—	19.57(72)	—	—	4.35(16)	—	—	44.02(162)
Suillus subaureus	—	—	32.07(118)	—	—	19.29(71)	—	—	4.35(16)	—	—	44.29(163)
Suillus variegatus	—	—	34.24(126)	—	—	19.84(73)	—	—	4.08(15)	—	—	41.85(154)
Suillus occidentalis	—	—	30.98(114)	—	—	19.02(70)	—	—	3.80(14)	—	—	46.2 (170)
Suillus placidus	—	—	32.80(122)	—	—	19.09(71)	—	—	4.03(15)	—	—	44.09(164)
Suillus paluster	—	—	32.78(119)	—	—	19.56(71)	—	—	3.03(11)	—	—	44.63(162)
Suillus lakei	—	—	32.41(117)	—	—	17.73(64)	—	—	4.43(16)	—	—	45.43(164)
Paxillus involutus	—	—	30.79(113)	—	—	19.89(73)	—	—	4.09(15)	—	—	45.23(166)
Paxillus ammoniavirescens	—	—	31.61(116)	—	—	19.62(72)	—	—	4.36(16)	—	—	44.41(163)
Boletus reticuloceps	—	—	31.25(115)	—	—	20.11(74)	—	—	3.53(13)	—	—	45.11(166)
Boletus reticuloceps	—	—	32.70(120)	—	—	19.35(71)	—	—	4.09(15)	—	—	43.87(161)
Rhizopogon vinicolor	—	—	32.34(119)	—	—	19.57(72)	—	—	4.35(16)	—	—	43.75(161)
Gyrodon lividus	—	37.48(539)	31.62(117)	—	14.74(212)	19.46(72)	—	4.87 (70)	4.59(17)	—	42.91(617)	44.32(164)
Xerocomus badius	—	36.86(530)	33.51(123)	—	14.53(209)	19.35(71)	—	4.66 (67)	4.09(15)	—	43.95(632)	43.05(158)
Suillus brevipes	—	37.22(533)	33.42(123)	—	14.53(208)	19.57(72)	—	4.89 (70)	4.89(18)	—	43.37(621)	42.12(155)
Neolentinus lepideus	—	36.65(520)	—	—	15.01(213)	—	—	4.79 (68)	—	—	43.55(618)	—
Heliocybe sulcata	—	37.34(531)	—	—	14.98(213)	—	—	4.78 (68)	—	—	42.90(610)	—
Gloeophyllum trabeum	—	37.12(529)	—	—	14.81(211)	—	—	4.84 (69)	—	—	43.23(616)	—
Laetiporus sulphureus	—	37.00(528)	—	—	14.86(212)	—	—	4.84 (69)	—	—	43.31(618)	—
Daedalea quercina	—	36.99(530)	33.06(122)	—	14.79(212)	18.70(69)	—	4.75 (68)	4.61(17)	—	43.48(623)	43.63(161)
Lentinus tigrinus	41.88(201)	38.31(547)	31.79(117)	10.83(52)	14.50(207)	19.84(73)	5.00(24)	4.41 (63)	3.53(13)	42.29(203)	42.79(611)	44.84(165)
Polyporus arcularius	42.29(203)	—	31.58(120)	12.08(58)	—	20.26(77)	6.04(29)	—	3.68(14)	39.58(190)	—	44.47(169)
Polyporus brumalis	42.29(203)	38.20(547)	30.53(116)	12.08(58)	14.73(211)	19.21(73)	6.04(29)	4.82 (69)	3.95(15)	39.58(190)	42.25(605)	46.32 176)
Trametes coccinea	42.54(208)	—	32.34(119)	12.88(63)	—	19.29(71)	6.34(31)	—	5.16(19)	38.24(187)	—	43.21(159)

Trametes versicolor	43.44(202)	—	32.00(120)	12.90(60)	—	18.93(71)	6.02(28)	—	2.93(11)	37.63(175)	—	46.13(173)
Trametes pubescens	—	—	30.13(113)	—	—	19.20(72)	—	—	5.33(20)	—	—	45.33(170)
Ganoderma boninense	44.82(212)	—	—	14.16(67)	—	—	6.77(32)	—	—	34.25(162)	—	—
Dichomitus squalens	43.33(208)	36.90(527)	30.62(113)	11.46(55)	15.13(216)	18.70(69)	5.42 26)	4.69 (67)	4.34(16)	39.79(191)	43.28(618)	46.34(171)
Stereum hirsutum	45.36(210)	37.68(537)	—	11.45(53)	14.67(209)	—	6.05(28)	4.56 (65)	—	37.15(172)	43.09(614)	—
Dentipellis sp.	—	37.38(530)	—	—	15.16(215)	—	—	5.08 (72)	—	—	42.38(601)	—
Russula ochroleuca	44.99(211)	36.82(524)	—	11.51(54)	14.90(212)	—	5.76(27)	4.78 (68)	—	37.74(177)	43.50(619)	—
Russula emetica	44.40(206)	38.12(539)	—	12.07(56)	14.07(199)	—	6.03(28)	4.81 (68)	—	37.50(174)	43.00(608)	—
Lactarius quietus	45.12(208)	36.93 524)	—	11.93(55)	14.73(209)	—	5.86(27)	4.65 (66)	—	37.09(171)	43.69(620)	—
Peniophora sp.	44.59(202)	37.80(533)	—	12.80(58)	14.89(210)	—	7.06(32)	4.89 (69)	—	35.54(161)	42.41(598)	—
Rickenella mellea	42.09(205)	—	—	12.73(62)	—	—	4.93(24)	—	—	40.25(196)	—	—
Pyrrhoderma noxium	45.01(212)	—	—	13.38(63)	—	—	6.16(29)	—	—	35.46(167)	—	—
Fomitiporia mediterranea	44.87(210)	—	—	12.39(58)	—	—	5.98(28)	—	—	36.75(172)	—	—
Sanghuangporus baumii	47.87(225)	—	—	12.55(59)	—	—	5.74(27)	—	—	33.83(159)	—	—
Schizopora paradoxa	44.54(216)	—	—	13.20(64)	—	—	5.15(25)	—	—	37.11(180)	—	—
Ramaria rubella	45.63(214)	—	—	11.73(55)	—	—	5.76(27)	—	—	36.89(173)	—	—
Auricularia subglabra	42.16(199)	—	—	12.50(59)	—	—	7.42(35)	—	—	37.92(179)	—	—
Exidia glandulosa	45.14(209)	—	—	12.53(58)	—	—	6.91(32)	—	—	35.42(164)	—	—
Cantharellus anzutake	44.52(199)	—	—	13.42(60)	—	—	6.49(29)	—	—	35.57(159)	—	—
Clavulina sp.	44.12(199)	36.68(522)	—	12.86(58)	14.90(212)	—	5.54(25)	5.13 (73)	—	37.47(169)	43.29(616)	—
Cryptococcus neoformans	—	—	29.62(117)	—	—	17.72(70)	—	—	4.56(18)	—	—	48.1 (190)
Cryptococcus wingfieldii	—	—	30.63(121)	—	—	17.97(71)	—	—	3.2913)	—	—	48.1 (190)
Cryptococcus gattii	—	—	32.15(127)	—	—	18.23(72)	—	—	3.54(14)	—	—	46.08(182)
Kwoniella heveanensis	—	—	30.38(120)	—	—	17.47(69)	—	—	4.30(17)	—	—	47.85(189)
Kwoniella mangroviensis	—	—	30.38(120)	—	—	18.73(74)	—	—	4.81(18)	—	—	46.08(182)
Kwoniella bestiolae	—	—	30.89(122)	—	—	17.47(69)	—	—	4.56(18)	—	—	47.09(186)

Kwoniella dejecticola	—	—	31.12(122)	—	—	17.09(67)	—	—	4.59(18)	—	—	47.19(185)
Apiotrichum porosum	—	—	31.59(121)	—	—	19.58(75)	—	—	4.96(19)	—	—	43.86(168)
Naematelia encephala	—	—	28.71(116)	—	—	18.56(75)	—	—	5.69(23)	—	—	47.03(190)
Trichosporon asahii	43.1 (200)	—	31.41(120)	12.72(59)	—	19.11(73)	5.82(27)	—	3.93(15)	38.36(178)	—	45.55(174)
Cutaneotrichosporon oleaginosum	43.28(203)	—	—	12.15(57)	—	—	6.82(32)	—	—	37.74(177)	—	—
Saitozyma sp.	42.62(208)	—	31.22(123)	10.66(52)	—	18.53(73)	5.53(27)	—	5.84(23)	41.19(201)	—	44.42(175)
Saitozyma podzolica	—	—	29.19(115)	—	—	17.77(70)	—	—	5.33(21)	—	—	47.72(188)
Phaffia rhodozyma	39.67(194)	37.62(541)	31.00(115)	13.29(65)	14.88(214)	19.41(72)	7.16(35)	5.08 (73)	4.31(16)	39.88(195)	42.42(610)	45.28(168)

Table S6. Sequences used for 3-D structure prediction and conservation analysis of binding sites of HCS, AAR, SDH, respectively.

HCS		AAR		SDH	
**Strains**	**Accession number**	**Strains**	**Accession number**	**Strains**	**Accession number**
Tricholoma matsutake	KAF8222095.1	*Armillaria gallica*	PBK98805.1	*Tricholoma matsutake*	KAF8223463.1
Cortinarius glaucopus	KAF8802540.1	*Armillaria solidipes*	PBK70829.1	*Mycena galopus*	KAF8213335.1
Pluteus cervinus	TFK72970.1	*Coprinellus micaceus*	TEB29768.1	*Marasmius fiardii*	KAF9270414.1
Pholiota molesta	KAF8199646.1	*Amanita rubescens*	KAF8345359.1	*Coprinopsis marcescibilis*	TFK30628.1
Pterula gracilis	TFK98677.1	*Pluteus cervinus*	TFK71908.1	*Moniliophthora roreri*	ESK98395.1
Coprinopsis marcescibilis	TFK27163.1	*Crucibulum laeve*	TFK37590.1	*Crassisporium funariophilum*	KAF8167912.1
Coprinopsis cinerea	XP_001836353.1	*Crassisporium funariophilum*	KAF8157006.1	*Crucibulum laeve*	TFK40972.1
Coprinellus micaceus	TEB35407.1	*Pholiota molesta*	KAF8180379.1	*Flammulina velutipes* *	QCX08356.1
Mycena galopus	KAF8207270.1	*Laccaria bicolor* *	XP_001879618.1	*Armillaria gallica*	PBL00798.1
Gymnopus androsaceus	KAE9408410.1	*Lyophyllum atratum*	KAF8067844.1	*Armillaria solidipes*	PBK77012.1
Flammulina velutipes *	AGG53996.1	*Tricholoma matsutake*	KAF8223431.1	*Suillus decipiens*	KAG2077096.1
Cylindrobasidium torrendii	KIY65962.1	*Gyrodon lividus*	KAF9226319.1	*Suillus clintonianus*	XP_041206159.1
Armillaria solidipes	PBK75543.1	*Xerocomus badius*	KAF8553785.1	*Suillus ampliporus*	KAG0709064.1
Armillaria gallica	PBL03758.1	*Suillus brevipes*	KAF3875411.1	*Suillus paluster*	XP_041183794.1
Trametes coccinea	OSD06237.1	*Neolentinus lepideus*	KZT28090.1	*Suillus brevipes*	KAG2754539.1
Trametes versicolor	EIW63959.1	*Heliocybe sulcata*	TFK49407.1	*Suillus occidentalis*	KAG1773126.1
Dichomitus squalens	XP_007359783.1	*Gloeophyllum trabeum*	XP_007867945.1	*Suillus placidus*	KAG1783737.1
Polyporus arcularius	TFK88646.1	*Laetiporus sulphureus*	KZT10254.1	*Suillus americanus*	KAG2044207.1
Polyporus brumalis	RDX51874.1	*Daedalea quercina*	KZT70440.1	*Suillus subalutaceus*	XP_041248104.1
Lactarius quietus	KAF8274751.1	*Lentinus tigrinus*	RPD65725.1	*Suillus subaureus*	XP_041199417.1
Russula ochroleuca	KAF8485787.1	*Polyporus brumalis*	RDX54732.1	*Suillus cothurnatus*	KAG2149799.1
Russula emetica	KAF8501388.1	*Dentipellis* sp.	KAA1473026.1	*Suillus lakei*	KAG1757371.1
Peniophora sp.	KZV64376.1	*Russula ochroleuca*	KAF8480159.1	*Suillus bovinus*	XP_041307276.1

Pyrrhoderma noxium	PAV16546.1	*Russula emetica*	KAF8491911.1	*Suillus variegatus*	KAG1834565.1
Ramaria rubella	KAF8591094.1	*Lactarius quietus*	KAF8271296.1	*Suillus tomentosus*	KAG1866257.1
Exidia glandulosa	KZV97461.1	*Peniophora* sp.	KZV74202.1	*Suillus hirtellus*	KAG2062054.1
Clavulina sp.	KAF8309935.1	*Clavulina* sp.	KAF8318757.1	*Suillus discolor*	XP_041298324.1
Cantharellus anzutake	KAF8339827.1	—	—	*Suillus plorans*	XP_041164869.1
Trichosporon asahii	XP_014181037.1	—	—	*Suillus fuscotomentosus*	XP_041220431.1
Phaffia rhodozyma	CDZ98541.1	—	—	*Xerocomus badius*	KAF8551619.1
—	—	—	—	*Gyrodon lividus*	KAF9229508.1
—	—	—	—	*Paxillus ammoniavirescens*	KAF8845261.1
—	—	—	—	*Polyporus brumalis*	RDX56030.1
—	—	—	—	*Polyporus arcularius*	TFK94363.1
—	—	—	—	*Lentinus tigrinus*	RPD63103.1
—	—	—	—	*Daedalea quercina*	KZT74771.1
—	—	—	—	*Trametes coccinea*	OSD06069.1
—	—	—	—	*Trichosporon asahii*	XP_014180072.1

Note: "*": Strains and amino acids used to predict 3D structure of proteins.

Table S7. The proteins with similar 3-D structures to the reported proteins of HCS, AAR and SDH in PDB database.

HCS in *F. velutipes*					AAR in *L. bicolor*					SDH in *F. velutipes*				
**PDB**	**TM-score**	**RMSD**	**IDEN**	**Cov**	**PDB**	**TM-score**	**RMSD**	**IDEN**	**Cov**	**PDB**	**TM-score**	**RMSD**	**IDEN**	**Cov**
6ktqA	0.837	1.09	0.355	0.847	4zxhA	0.754	1.82	0.203	0.765	2qrlA	0.984	0.35	0.543	0.986
6e1jA	0.786	1.91	0.276	0.821	6mfzA	0.538	3.66	0.237	0.581	1pjcA	0.747	3.36	0.168	0.870
3figB	0.698	3.35	0.156	0.769	2vsqA	0.511	5.70	0.160	0.598	1f8gA	0.709	3.38	0.179	0.829
4ov4A	0.683	3.15	0.221	0.754	6n8eA	0.500	5.20	0.162	0.577	1m2wA	0.430	5.67	0.090	0.630
3ivtB	0.658	3.35	0.694	0.735	4zxjA	0.452	4.70	0.170	0.507	1gz3A	0.426	4.70	0.107	0.554
3bliA	0.623	1.65	0.252	0.649	5u89A	0.356	6.39	0.155	0.425	1p0fA	0.418	5.47	0.106	0.584
3a9iA	0.619	2.54	0.545	0.659	1ry2A	0.346	3.89	0.156	0.375	1hrkA	0.416	5.50	0.081	0.601
3rmjA	0.609	1.96	0.268	0.638	2d1rA	0.345	3.31	0.153	0.368	1pl6A	0.406	5.45	0.117	0.565
6ndsA	0.565	2.61	0.185	0.616	1amuA	0.343	2.62	0.260	0.357	1u1uA	0.404	6.37	0.033	0.644
2cw6A	0.553	2.25	0.203	0.595	1mdbA	0.337	3.12	0.178	0.356	1gcaA	0.402	5.47	0.070	0.573

Note: "PDB": Protein Data Bank database. "TM-score": Template Modeling score. "RSMD": RMSD is the RMSD between residues that are structurally aligned by TM-align. "IDEN": IDEN is the percentage sequence identity in the structurally aligned region. "Cov": Cov represents the coverage of the alignment by TM-align.

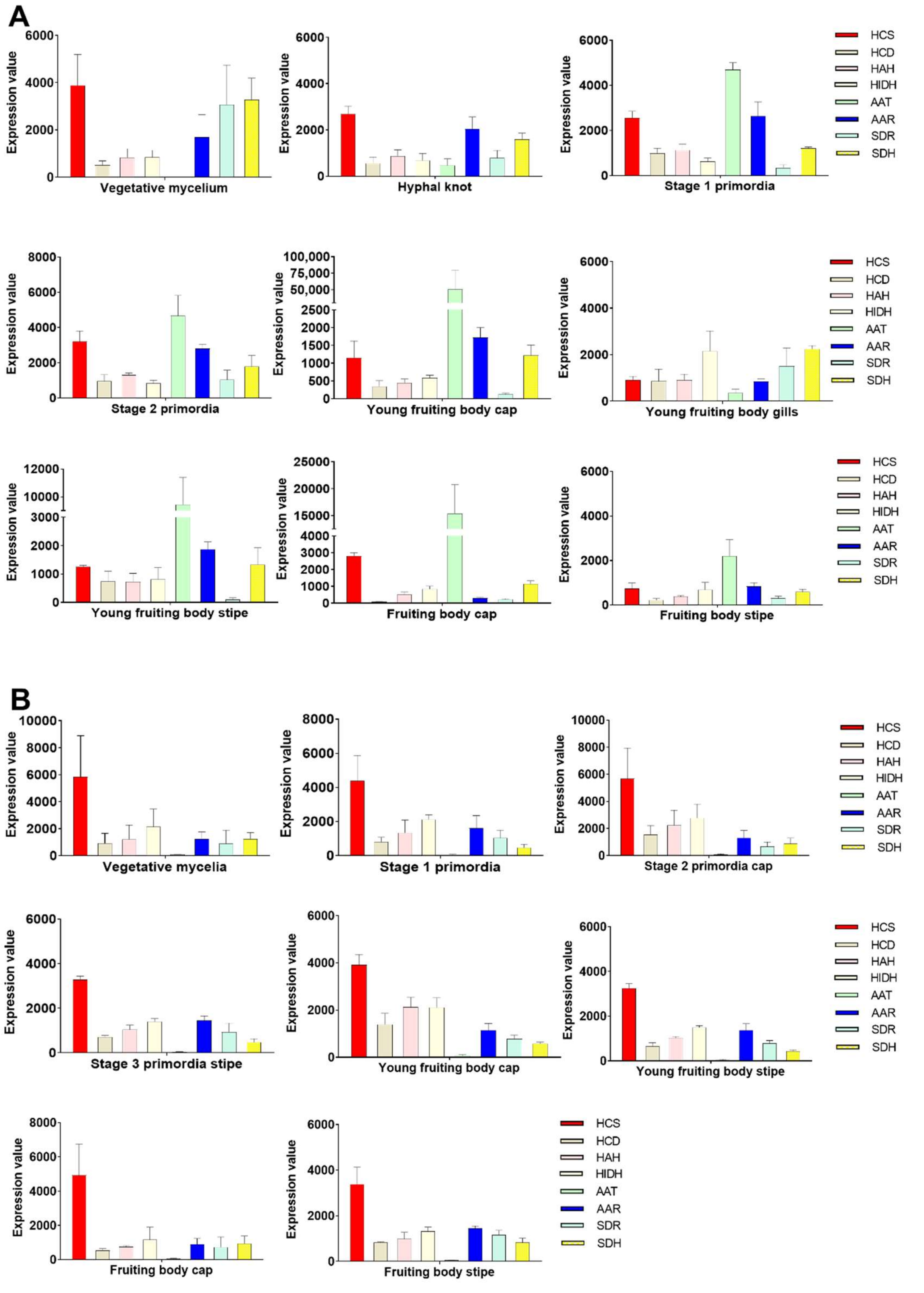

Figure S1. Expression levels of the genes of eight enzymes involved in AAA pathway in different development stages by RNA-seq analysis in *Coprinopsis cinerea* and *Lentinus tigrinus*. (A) Expression levels of the genes in different development stage in *C. cinerea.* (B) Expression levels of the genes in different development stage in *L. tigrinus.* (The RNA-seq data are from K. Krizsan et al. 2019)

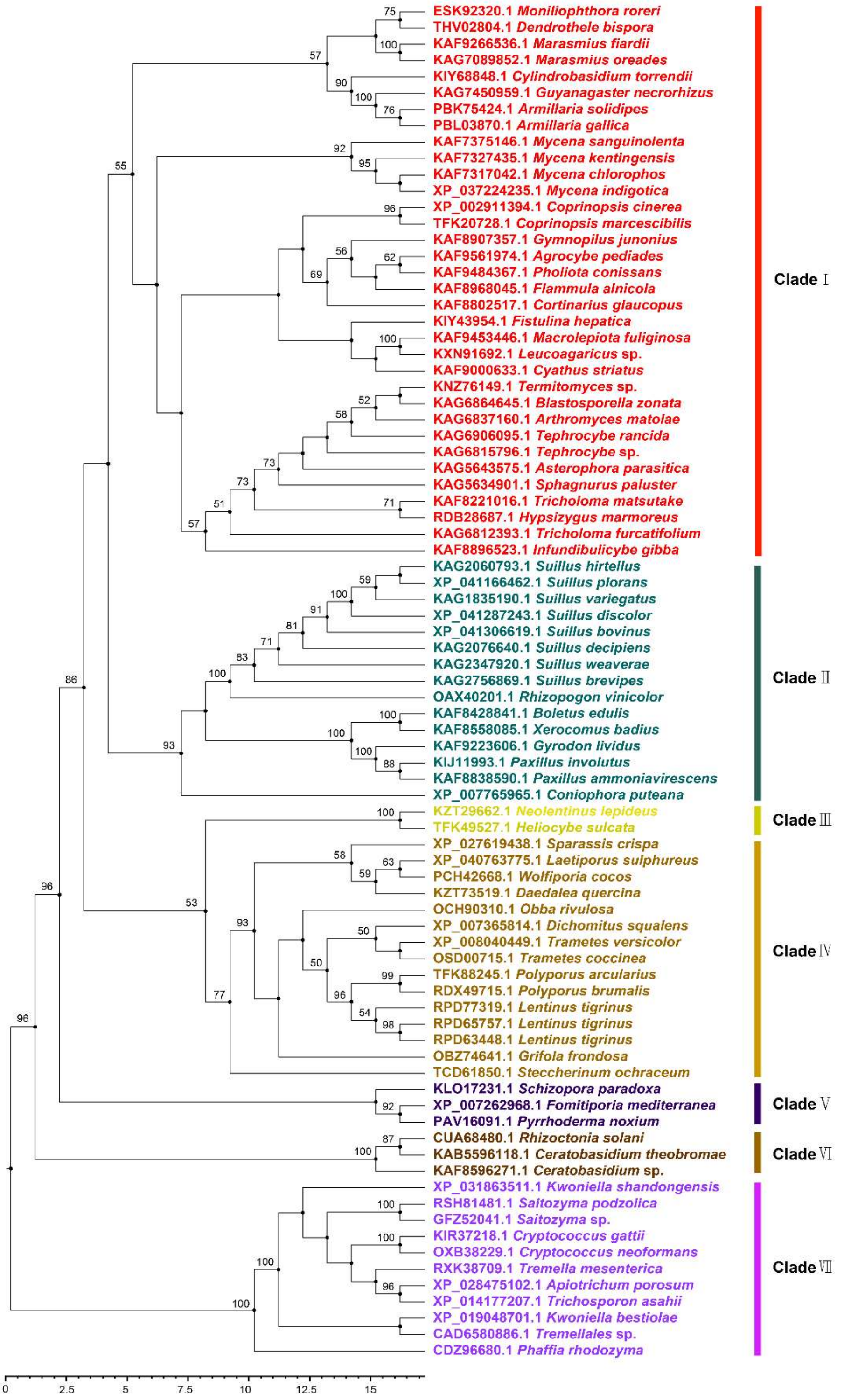

Figure S2. Phylogenetic tree of HAH in Agaricomycetes with Tremellomycetes as outgroup. MP bootstrap values (≥50%) of each clade are indicated at nodes. Scale bar in the upper left indicates substitutions per site. (Clade Ⅰ: Agaricales; Clade Ⅱ: Boletales; Clade Ⅲ: Gloeophyllales; Clade Ⅳ: Polyporales; Clade Ⅴ: Hymenochaetales; Clade Ⅵ: Cantharellals; Clade Ⅶ: Tremellomycetes)

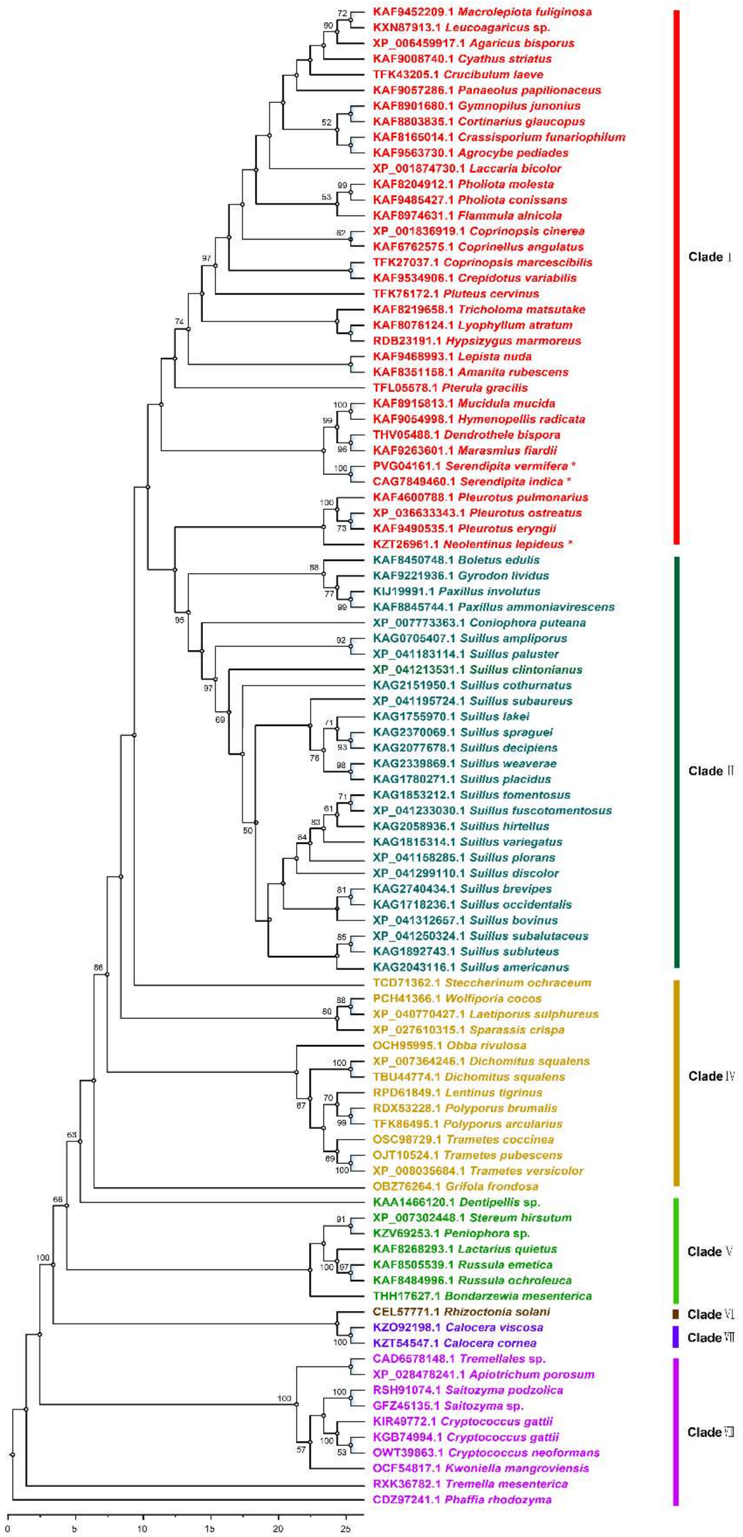

Figure S3. Phylogenetic tree of HIDH in Agaricomycetes with Tremellomycetes as outgroup. MP bootstrap values (≥50%) of each clade are indicated at nodes. Scale bar in the upper left indicates substitutions per site. (Clade Ⅰ: Agaricales; Clade Ⅱ: Boletales; Clade Ⅲ: Polyporales; Clade Ⅳ: Russulales; Clade Ⅴ: Cantharellals; Clade Ⅵ: Dacrymycetes; Clade Ⅶ: Tremellomycetes)

A

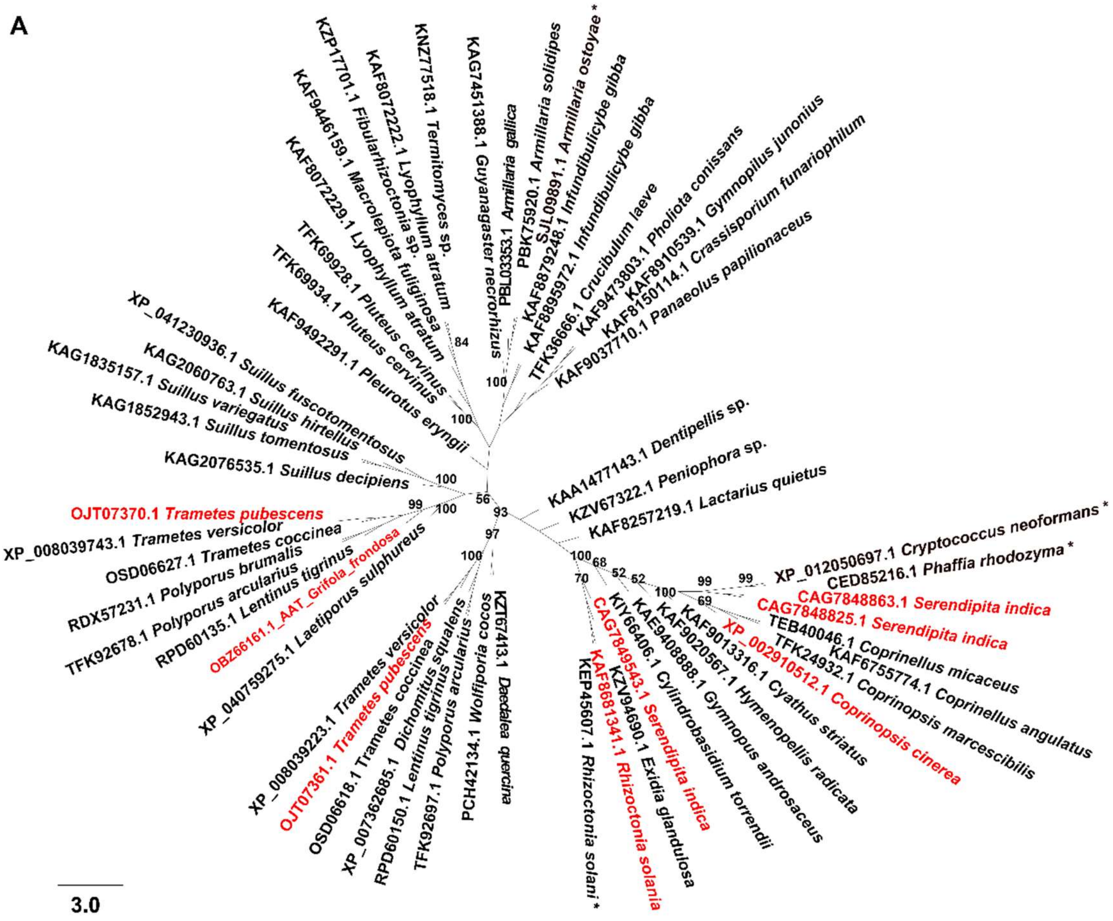

B

Olders	Strains	Family	Accession
Agaricales	*Coprinopsis cinerea*	MocR family	XP_002910512.1
Polyporales	*Trametes pubescens*	MocR family	OJT07370.1
Polyporales	*Trametes pubescens*	MocR family	OJT07361.1
Polyporales	*Grifola frondosa*	MocR family	OBZ66161.1
Cantharellales	*Rhizoctonia solani*	MocR family	KAF8681341.1
Sebacinales	*Serendipita indica*	MocR family	CAG7849543.1
Sebacinales	*Serendipita indica*	MocR family	CAG7848825.1
Sebacinales	*Serendipita indica*	MocR family	CAG7848863.1

Figure S4. Phylogenetic tree of AAT in Agricomycetes. (A). ML tree based on AAT amino acid sequences of 59 strains. MP bootstrap values (≥50%) of each clade are indicated at nodes. Scale bar in the upper left indicates substitutions per site. (B). The reported α–aminoadipate aminotransferases in Agaricomycetes.

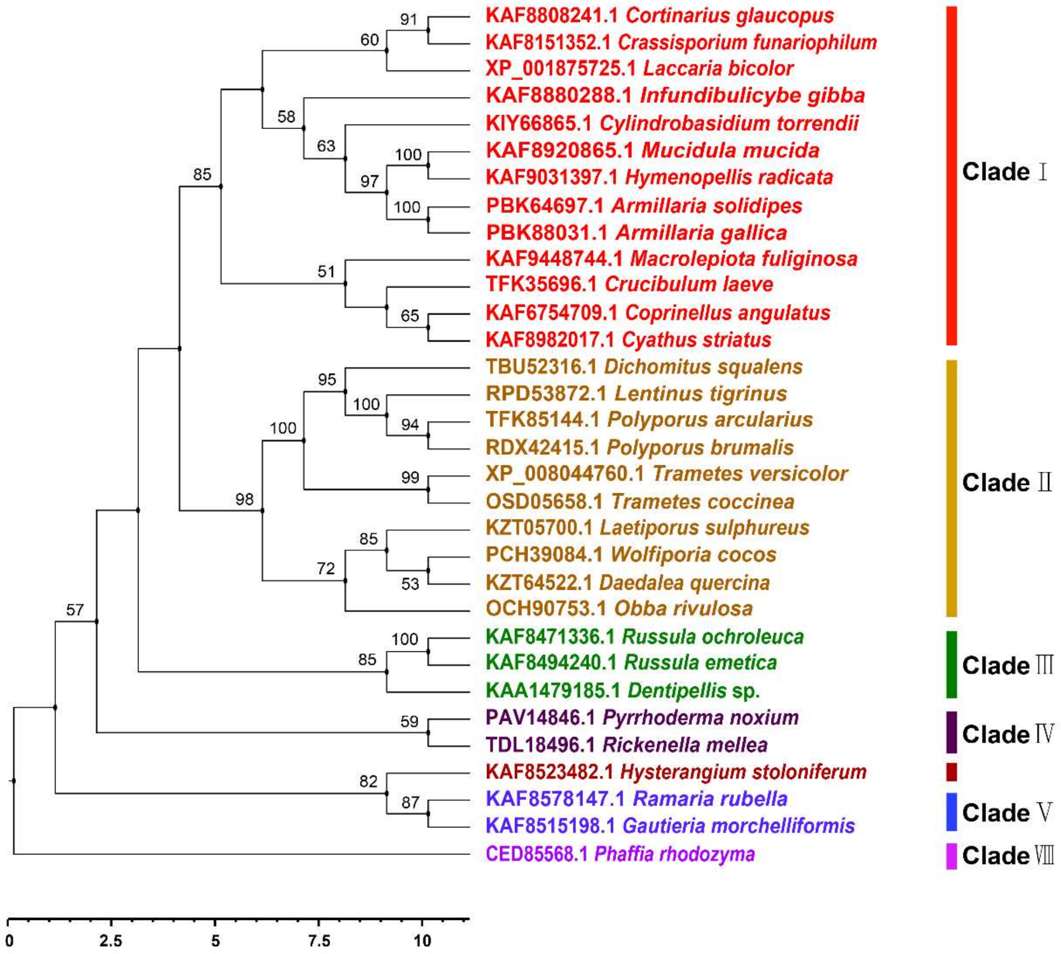

Figure S5. Phylogenetic tree of SDR in Agaricomycetes with Tremellomycetes as outgroup. MP bootstrap values (≥50%) of each clade are indicated at nodes. Scale bar in the upper left indicates substitutions per site. (Clade I: Agaricales; Clade II: Polyporales; Clade III: Russulales; Clade IV: Hymenochaetales; Clade V: Hysterangiales; Clade VI: Gomphales; Clade VII: Tremellomycetes)

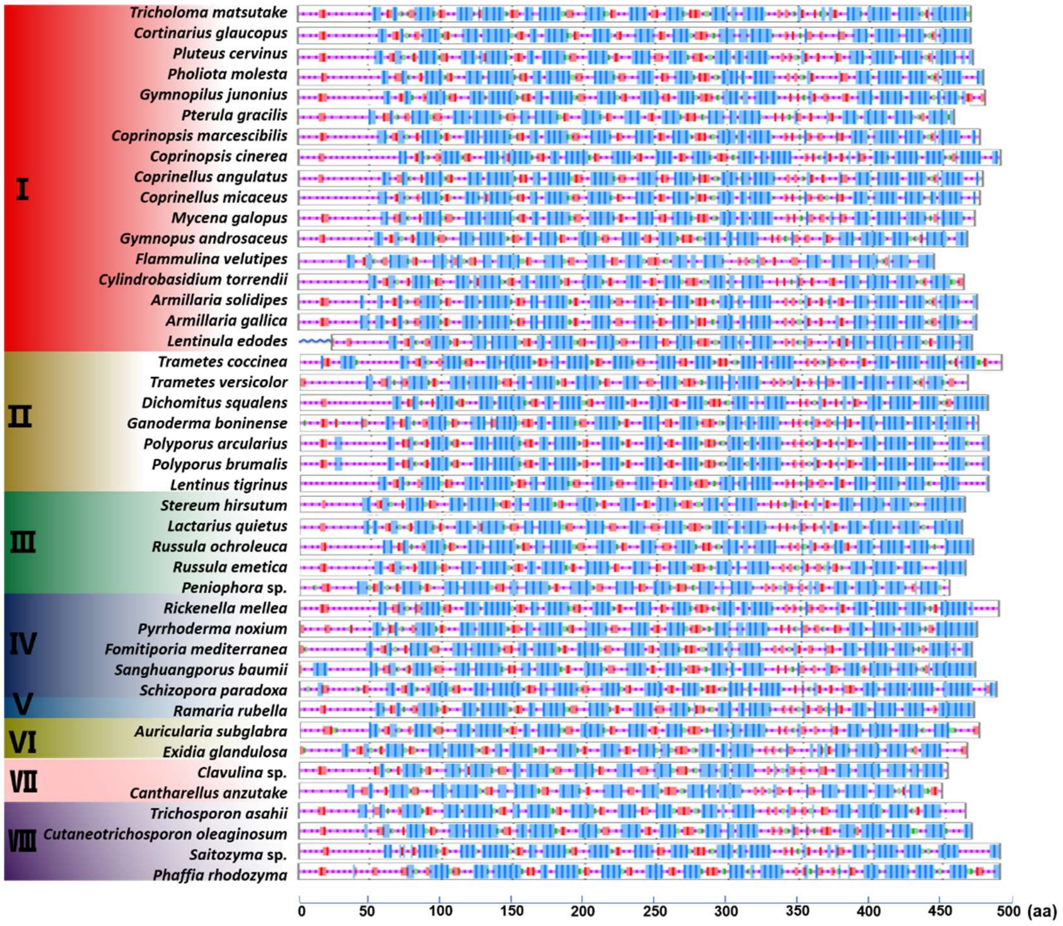

Figure S6. Characteristics of the secondary structures of HCS in Agaricomycetes. "Blue: Alpha-helix, Red: Extended strand, Green: Beta turn, Golden: Random coil" (I: Agaricales, II: Polyporales, III: Russulales, IV: Hymenochaetales, V: Gomphales, VI: Auriculariales, VII: Cantharellals, VIII: Tremellomycetes).

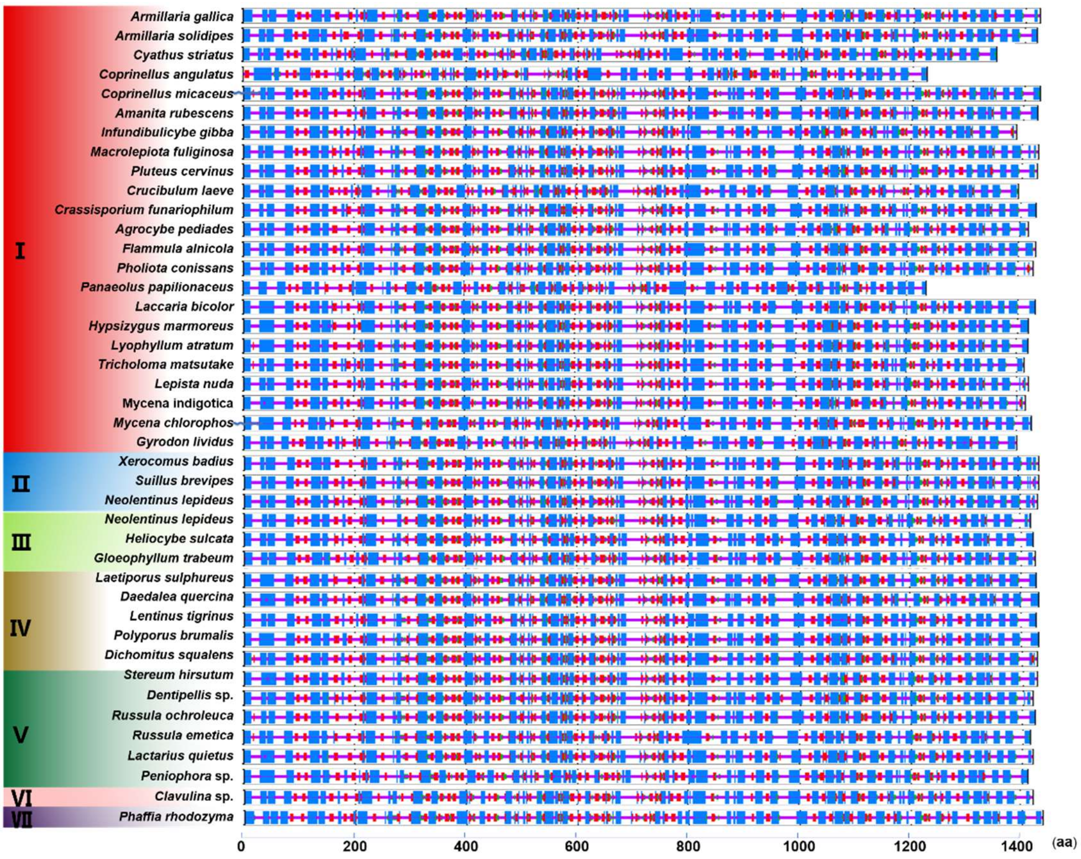

Figure S7. Characteristics of the secondary structure of AAR in Agaricomycetes. "Blue: Alpha-helix, Red: Extended strand, Green: Beta turn, Golden: Random coil" (I: Agaricales, II: Boletales, III: Gloeophyllales, IV: Polyporales, V: Russulales, VI: Cantharellals, VII: Tremellomycetes)

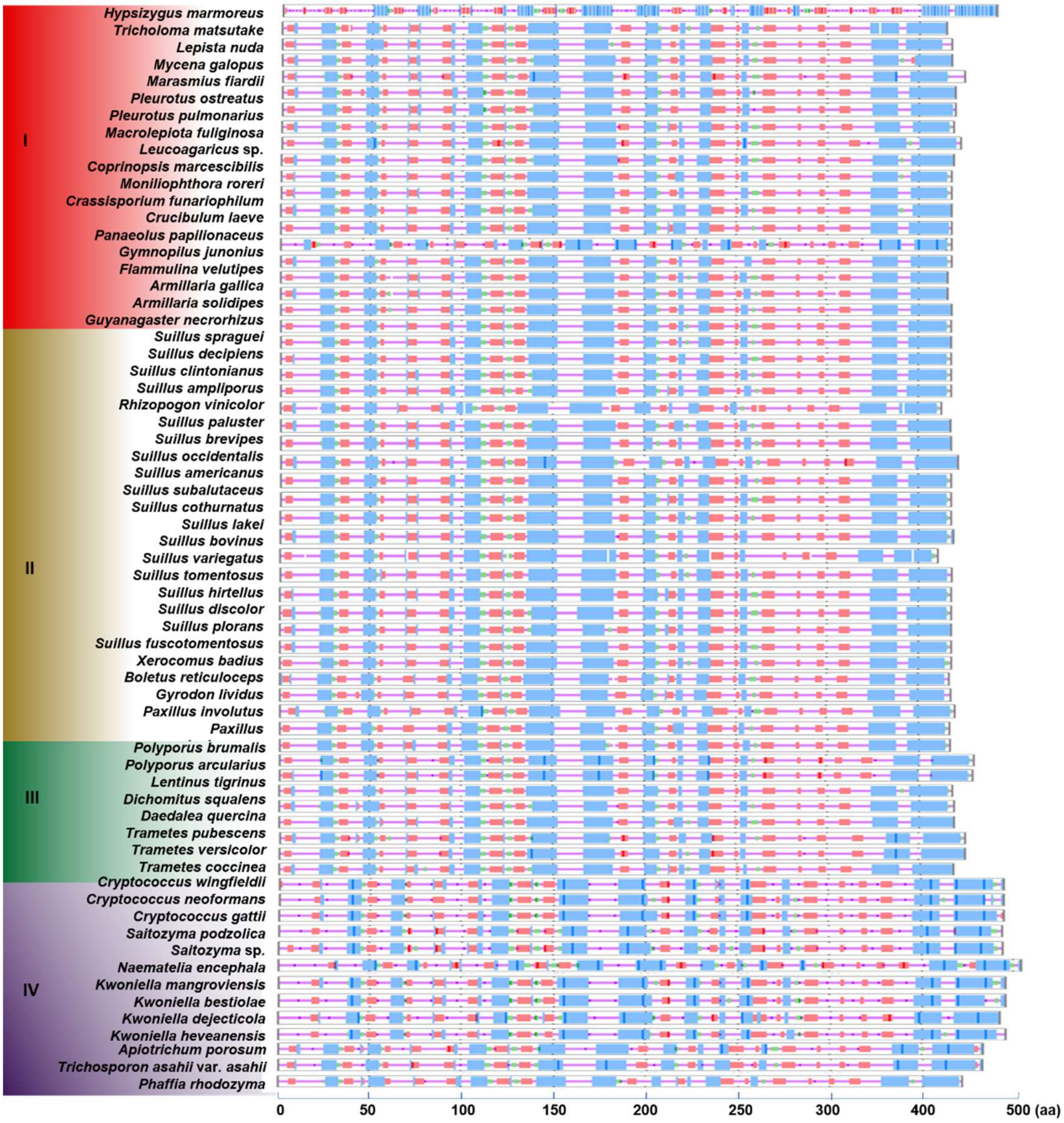

Figure S8. Characteristics of the secondary structure of SDH in Agaricomycetes. "Blue: Alpha-helix, Red: Extended strand, Green: Beta turn, Golden: Random coil" (I: Agaricales, II: Boletales, III: Polyporales, IV: Tremellomycetes)

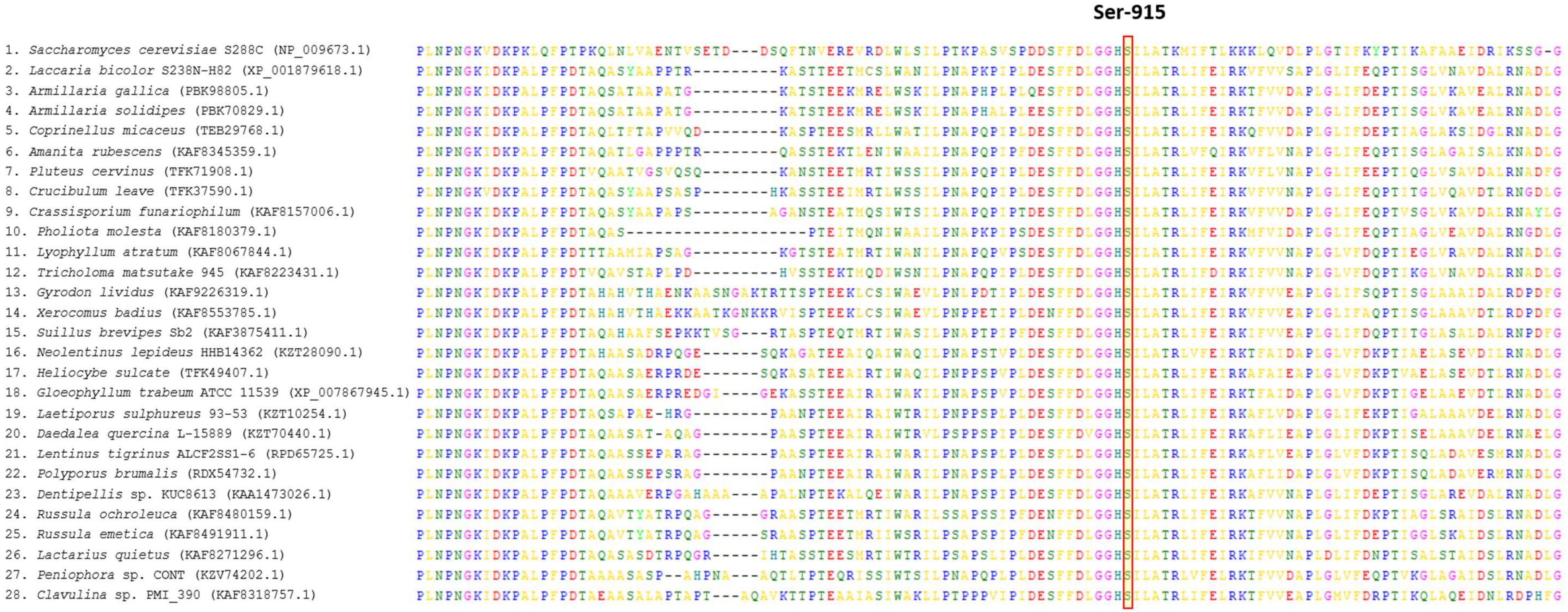

Figure S9. Multiple sequence alignment analysis of PCP domain in AAR from *Saccharomyces cerevisiae* with PCP domains from 27 species in Agaricomycetes.
